# Identifying Ligand Binding Conformations of the β2-Adrenergic Receptor by Using Its Agonists as Computational Probes

**DOI:** 10.1371/journal.pone.0050186

**Published:** 2012-12-31

**Authors:** Basak Isin, Guillermina Estiu, Olaf Wiest, Zoltán N. Oltvai

**Affiliations:** 1 Department of Pathology, University of Pittsburgh, Pittsburgh, Pennsylvania, United States of America; 2 Department of Chemistry and Biochemistry and Center for Rare and Neglected Diseases, University of Notre Dame, Notre Dame, Indiana, United States of America; 3 Laboratory of Computational Chemistry and Drug Design, Laboratory of Chemical Genomics, Peking University, Shenzhen Graduate School, Shenzhen, China; Monash University, Australia

## Abstract

Recently available G-protein coupled receptor (GPCR) structures and biophysical studies suggest that the difference between the effects of various agonists and antagonists cannot be explained by single structures alone, but rather that the conformational ensembles of the proteins need to be considered. Here we use an elastic network model-guided molecular dynamics simulation protocol to generate an ensemble of conformers of a prototypical GPCR, β_2_-adrenergic receptor (β_2_AR). The resulting conformers are clustered into groups based on the conformations of the ligand binding site, and distinct conformers from each group are assessed for their binding to known agonists of β_2_AR. We show that the select ligands bind preferentially to different predicted conformers of β_2_AR, and identify a role of β_2_AR extracellular region as an allosteric binding site for larger drugs such as salmeterol. Thus, drugs and ligands can be used as “computational probes” to systematically identify protein conformers with likely biological significance.

## Introduction

One of the best examples for illustrating the conformational diversity of biomolecules is the superfamily of G protein-coupled receptors (GPCR), the largest known superfamily of cell surface receptors. GPCRs are involved in a number of important ligand-receptor interactions, including responses to light, odorant molecules, neurotransmitters, hormones, and a variety of other signals. Each active GPCR can stimulate hundreds of G-proteins that represents the first amplification step in the GPCR signaling cascade [1]. Additionally, cytoplasmic proteins such as kinases and arrestins also interact with active GPCRs for signal quenching to prevent persistent receptor stimulation [2]. The fact that ∼30% of all approved drugs target GPCRs highlights their pharmacological importance [3].

Insights into the conformational states and the complexity of GPCR dynamics have been obtained from a range of experiments, which revealed that the activation of GPCRs is a multistep process, and even at basal activity levels the receptors display different conformations. Furthermore, different active state conformations of GPCRs can be stabilized by different agonists, which results in their association with different downstream signaling molecules [4]–[11].

**Figure 1:**
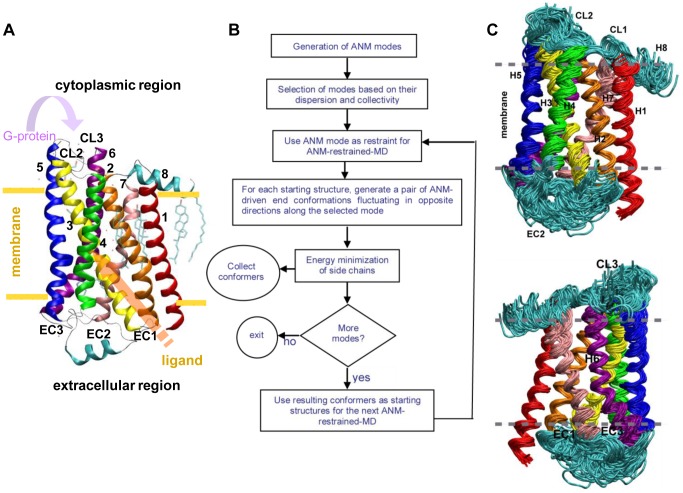
Protocol for ANM-restrained-MD simulations and β_2_AR conformations. **A.** Structure of β_2_AR. Transmembrane helices 1–7 are labeled by numbers and colored in red, orange, yellow, green, blue, purple, and pink, respectively. Cytoplasmic helix 8 and the short extracellular helix below the binding cavity in extracellular loop 2 are colored in cyan. Ligand- and G-protein binding sites are shown by arrows. The palmitolyl group that is anchored to the membrane from the H8 is also shown in cyan. **B.** The protocol for generating the ensemble of conformations by ANM-restrained-MD algorithm. **C.** Ribbon diagrams of β_2_AR conformations. Front view (top) and back view (bottom) of β2AR conformers generated by ANM-restrained-MD are shown.

A prototypical GPCR, β_2_-adrenergic receptor (β_2_AR), is crucial for the physiological regulation of cardiovascular and pulmonary functions through the binding of catecholamines, such as epinephrine or norepinephrine. β_2_AR comprises cytoplasmic (CP), transmembrane (TM), and extracellular (EC) domains (Fig. 1A, Fig. S1A) and contains a bundle of seven trans-membrane (TM) helices (H1–H7). The CP domain of β_2_AR contains three cytoplasmic loops, CL1–CL3, that connects helices H1–H2, H3–H4, and H5–H6, respectively. The EC domain consists of the N-terminus and three interhelical extracellular loops (EC1–EC3) between TM helices H2–H3, H4–H5 and H6–H7. EC2 (between H4 and H5) contains a short helix at a position above the ligand binding cavity at the TM region (Fig. 1A, Fig. S1A).

Available experimental evidence indicates that GPCRs have several conserved “microdomains”. The allosteric disruptions of specific contacts and conformational changes at these sites are crucial for GPCR activation [12]. These microdomains include (i) the highly conserved (D)ERY motif at the CP end of H3 and the X_1_BBX_2_X_3_B motif at the CP end of H6 (B, basic; X, non-basic) [1], [13], (ii) the Asn-Asp pair in H1 and H2, and the NPXXY motif in H7, respectively [14], [15], and (iii) the aromatic cluster surrounding the ligand binding pocket, including the rotamer toggle [16], [17]. Recent publications of several GPCR crystal structures have in turn prompted several computational studies. While much attention has been paid to the G-protein binding motifs at the cytoplasmic region, particularly to the (D)ERY motif and the salt bridge at the CP sites of H3 and H6 [18]–[22], the global dynamics of the entire protein that leads to its activation and the functional roles of its extracellular sites have only been explored more recently [23], [24].

We previously developed a multiscale protocol called Anisotropic Network Model (ANM) [25], [26] restrained Molecular Dynamics (MD) [24]. This protocol allows for the sampling of long time-scale, biologically-relevant global motions of a biomolecular system with realistic deformations in the presence of the protein's explicit environment. We previously applied ANM-restrained-MD to the first structural representative of GPCRs, rhodopsin, to study its activation mechanism and the interactions with its ligands [24]. Here, we use the ANM-restrained-MD protocol to generate conformational ensembles of β_2_AR. Along with the existing crystal structures, these conformational ensembles are probed for binding modes of β_2_AR by docking its known agonists against them. Our simulations reveal that, in agreement with previous experimental data, β_2_AR alters its ligand binding site and residues that are critical for ligand binding and activation to make them accessible for drugs. They also show that agonists with distinct structures (such as salmeterol and epinephrine [Fig. S2]) bind to distinct conformations of β_2_AR. Thus, our simulations uncover the novel ligand-selected conformers of β_2_AR that may engage the distinct sets of interacting proteins for initiating downstream signaling events, and suggest a generic scheme in which drugs and innate ligands are used as ‘computational probes’ to identify potential biological conformers of their binding proteins.

## Results

### ANM-restrained-MD generated conformations of β_2_AR

We used an ANM-restrained-MD protocol to generate conformational ensembles of β_2_AR (Fig. 1B, C). As discussed in the Materials and Methods section, the lowest 9 modes of ANM were followed in the plus and minus direction using ANM-restrained-MD. Figure S3A shows the time evolution of RMSD in C^α^ positions from the initial structure along the nine lowest modes that are used as targets in the MD simulations. The two curves in each graph refer to the opposite direction deformations. By the end of each simulation the conformation departs from the original one by an RMSD of about 1.5 Å. Figure S3B shows the time evolution of RMSD in C^α^ positions from the initial structure along the modes that are used as targets in the ANM-restrained-MD simulations recursively. Ribbon diagrams of select β_2_AR conformations obtained by these simulations are shown in Figure 1C (see Text S1 and Figure S3 for additional details).

**Figure 2:**
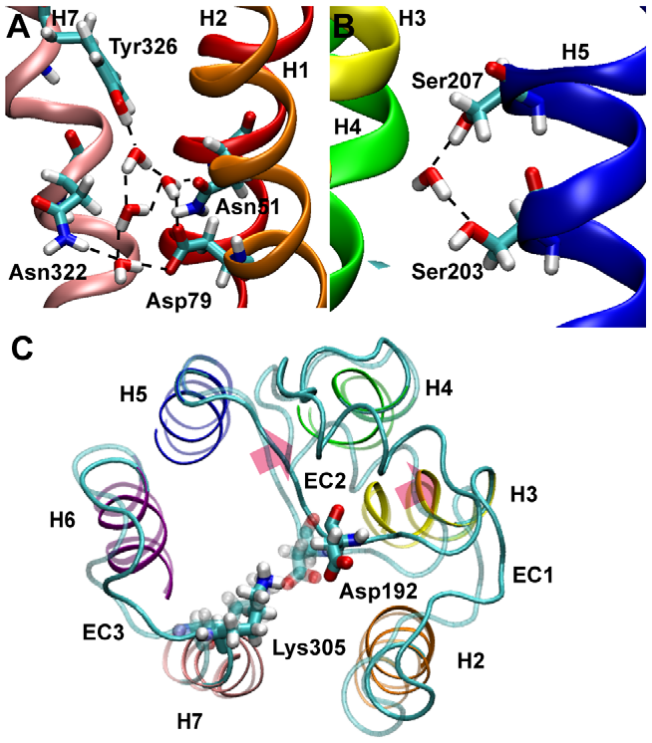
Microdomains of β_2_AR stabilized by new interactions and water molecules. **A**. Water molecules stabilizing the conserved NPXXY motif (left) and the Asn-Asp pair (right) within the transmembrane region, and (**B**) the critical catecholamine binding Ser203 and Ser207 residues at H5 in conformations where they both point to the ligand binding pocket and connected through a water molecule. **C**. The motion of extracellular loop two (EC2) and the residues that form the salt bridge at the EC site. The motion found by ANM-restrained-MD to break the salt bridge at the extracellular site and the opening of the extracellular site is shown. ANM-restrained-MD conformation and the carazolol-bound structures are in solid and transparent colors, respectively. The side chains of Asp192 at EC2 and Lys305 at H7 that form the salt bridge at the inactive state of β_2_AR are displayed on both structures. The motion of the EC2 including the short helix is depicted by red arrows.

Next, we analyzed the motions of β_2_AR microdomains (Fig. 2, Fig. S4) in the conformations that have been obtained by ANM-restrained-MD simulations. In the conformational ensemble, we find that the hydrogen bond network among H1 and H2 and H7 has the highest rigidity at the cytoplasmic side, mainly due to the water molecules that exist between the N-D pair and the NPXXY motif on H7 coordinating the H-bond network. Two residues that belong to the NPXXY motif at H7, Tyr326 and Asn322 are connected to Asn51 on H1 and Asp79 at H2 through four water molecules located in the cavity between H1, H2 and H7 (Fig. 2A).

Previous experimental evidence demonstrated that at least two of the three serine residues at H5 (Ser203, Ser204 and Ser207) are important for the binding of agonists with the catechol moiety. Only Ser203 is accessible to the binding pocket in the carazolol-bound inactive form of β_2_AR [27]. Our simulations show that the transposition of H5 with 1.5 Å toward the ligand binding pocket is sufficient for the accessibility of all three serine residues in the binding pocket. The simulations also reveal that in the absence of ligand a water molecule migrates and interacts with Ser203 and Ser207 (Fig. 2C).

At the EC site of β_2_AR, EC2 and H7 are connected with a salt bridge between Asp192-Lys305 [4]. During ANM-guided-MD simulations, the distance between Asp192 and Lys305 ranges from 1.8 Å to 4.3 Å. Figure 2C shows the extracellular site of the carazolol-bound inactive crystal structure superimposed on the ANM-restrained-MD conformation obtained by steering along the eighth mode of ANM in MD simulations. The residues Asp192 at EC2 and Lys305 at H7 are displayed in both structures (Fig. 2C). In the carazolol-bound inactive β_2_AR structure Asp192-Lys305 forms a salt bridge with a distance of 1.8 Å. In the conformation generated by the ANM-restrained MD this salt bridge is broken and the distance is increased to 3.5 Å. Furthermore, the entire EC2, including the short helix that occupies a position below the ligand binding site moves away from EC3 as well as from the ligand binding pocket. This indicates that the EC site is flexible for ligand diffusion and accommodates the ligands by coupling to the ligand binding site.

### Conformations of binding site residues

As it is detailed in the Methods section (see also Fig. S5), we used the XCluster module of the Schrodinger suite [28] for choosing the representative conformations that the agonists were docked against. The root mean square deviation (RMSD) of the residues that have been shown previously to be critical for the ligand binding and activation of the protein were used as the clustering criteria. The conformations of the residues are displayed on the structures that are selected by the XCluster, shown in Figure S6.

### ANM-restrained MD conformations that accommodate salmeterol and epinephrine

After choosing the representative conformations by Xcluster, we docked the agonists against them using the Glide SP and XP as incorporated in the Schrodinger Suite [29], [30]. The resulting complexes were selected based on both their GlideXP scores and their poses, i.e. the geometries of the agonist-protein interactions that had been shown to be critical for ligand binding and protein activation (see Table 1 for β_2_AR agonist binding residues and the agonist motives that are known to bind them) [10], [16], [17], [31]–[39]. This allows the selection of ANM-restrained-MD generated β_2_AR conformations that bind most favorable to known agonists. For example, the most favorable β_2_AR-salmeterol complex derives from the conformation that was obtained by steering the ANM-restrained-MD along the eighth mode in the minus direction (8M). Similarly, the most favorable β_2_AR-epinephrine complex is derived from the conformation that is found by following the second mode in plus direction (2P) in the ANM-restrained MD simulations. The β_2_AR-formoterol complex has been obtained by an ANM-restrained-MD conformation that is obtained by a combination of modes (first mode in plus, second mode in minus and third mode in minus directions) by using the algorithm described in the methods section (Figs. S3B and S10). It should be noted that the starting structures of the ANM-restrained-MD simulations do not give good scores or poses for any of the three known agonists, which is to be expected considering the fact that they are derived from the crystal structure of the inactive form of β_2_AR. To show the similarity of the modes (the ANM-restrained-MD targets) to the resulting ANM-restrained MD conformations we have calculated the RMSD per residue between them. Figure S7 shows the RMSD per residue graphs and ribbon diagrams for 8M and 2P and their target ANM modes. The left panel shows RMSD profiles of C^α^ as a function of residue index, reached by the ANM-restrained MD protocol using modes 8 in minus (8M) and 2 in plus (2P) directions. The right panels show ribbon diagrams of 8M (A) and 2P (B) β_2_AR conformations. As it can be observed from Figure S7 both of the ANM-restrained MD conformations that form complexes with epineprine and salmeterol reach their corresponding target ANM modes.

**Table 1:** β_2_AR residues that are shown to be interacting with agonists.

Helix	Residue	Agonist motifs	Reference
**H3**	Asp113, Val114	β-OH	Strader et al., 1988; Chung et al., 1988; Wieland et al., 1996; Balmforth et al., 1997; Swaminath et al., 2005; Arakawa et al., 2009
**H5**	Ser203, Ser204, Ser207, Pro211	Head group OHs	Strader et al., 1989; Liapakis et al., 2000, Swaminath et al., 2005
**H6**	Phe282, Trp286, Phe289, Asn293, Asn297	Head group (Phe 282, Trp286, Phe289), Protonated amine (Asn293)	Strader et al., 1994; Cho et al., 1995; Javitch et al., 1998; Shi et al., 2002; Chen et al., 2002; Swaminath et al., 2005
**H7**	Tyr308, Asn312, Try316	Ethanolamine tail	Huber et al., 2008; Ahuja & Smith, 2009

To compare the sizes and the locations of epinephrine, salmeterol and BI-167107 (the insoluble agonist crystallized in the active structures of β_2_AR) [22], [40] in the protein-ligand complexes, we examined the molecular surfaces of these ligands using MOE (Molecular Operating Environment, Chemical Computing Group Inc.). Ribbon diagrams of β_2_AR conformations are shown in gray in Figure S8. The head groups of all agonist are located at the orthosteric binding pocket and they interact with the Serine residues at H5 that are rotated towards the binding pocket in the ANM-restrained-MD conformations. While epinephrine populates only the orthosteric binding site both BI-167107 and salmeterol protrudes to the extracellular region.

**Figure 3:**
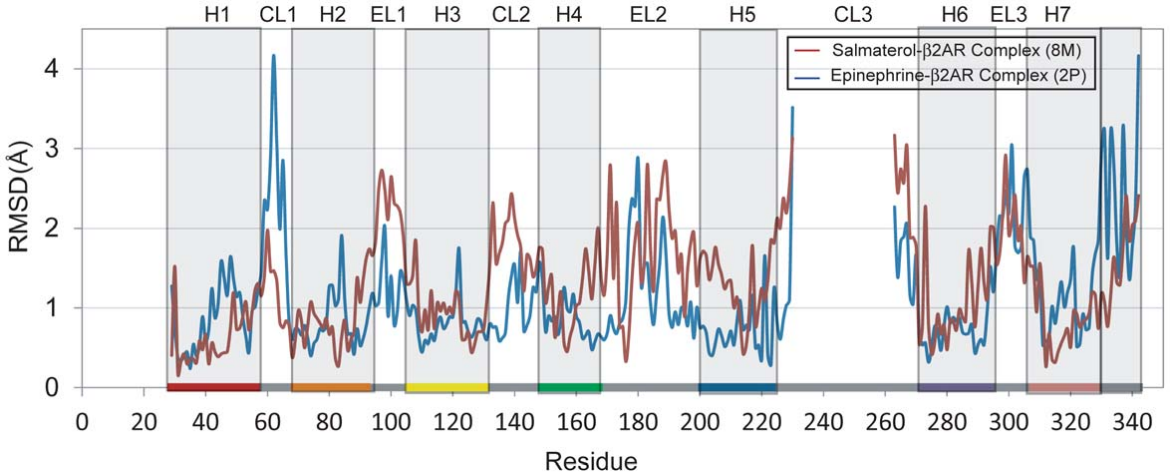
RMSD profiles as a function of residue index for the ANM-restrained-MD conformations that accommodate salmeterol and epinephrine. Red and blue curves represent RMSD per residue between the starting structure and the conformations that accommodate salmeterol and epinephrine, respectively. In both complexes, not only cytoplasmic region that binds to G-protein but also the extracellular region exhibits higher motilities. Although the overall RMSD profile of the extracellular region tends to maintain similar features in both conformations, 8M has larger structural rearrangements at EC2 and at the extracellular end of the helices that are connected by this loop. These structural arrangements lead to the accommodation of salmeterol at the extracellular region. The sequence ranges of the helices (H1–H8) are indicated by the labels on the upper abscissa and distinguished by gray bands. The color code of the helices that are used in the ribbon diagrams of β_2_AR are also displayed as colored bands at the bottom of the graph.

To further analyze the complexes we generated RMSD per residue graphs between the starting structures and the ANM-restrained-MD conformations (Fig. 3). In both complexes, not only the cytoplasmic region that bind to G-protein (CL2, CL3 and the cytoplasmic ends of the connecting helices) but also the extracellular region (including the loops EC1, EC2 and EC3) exhibit higher mobility. Although the overall RMSD profile of the extracellular region tends to maintain similar features in both conformations, 8M has larger structural rearrangements at EC2 and at the extracellular end of the helices that are connected by this loop. These structural arrangements lead to the accommodation of salmeterol at the extracellular region.

### Binding of the long-acting agonist, salmeterol

**Figure 4:**
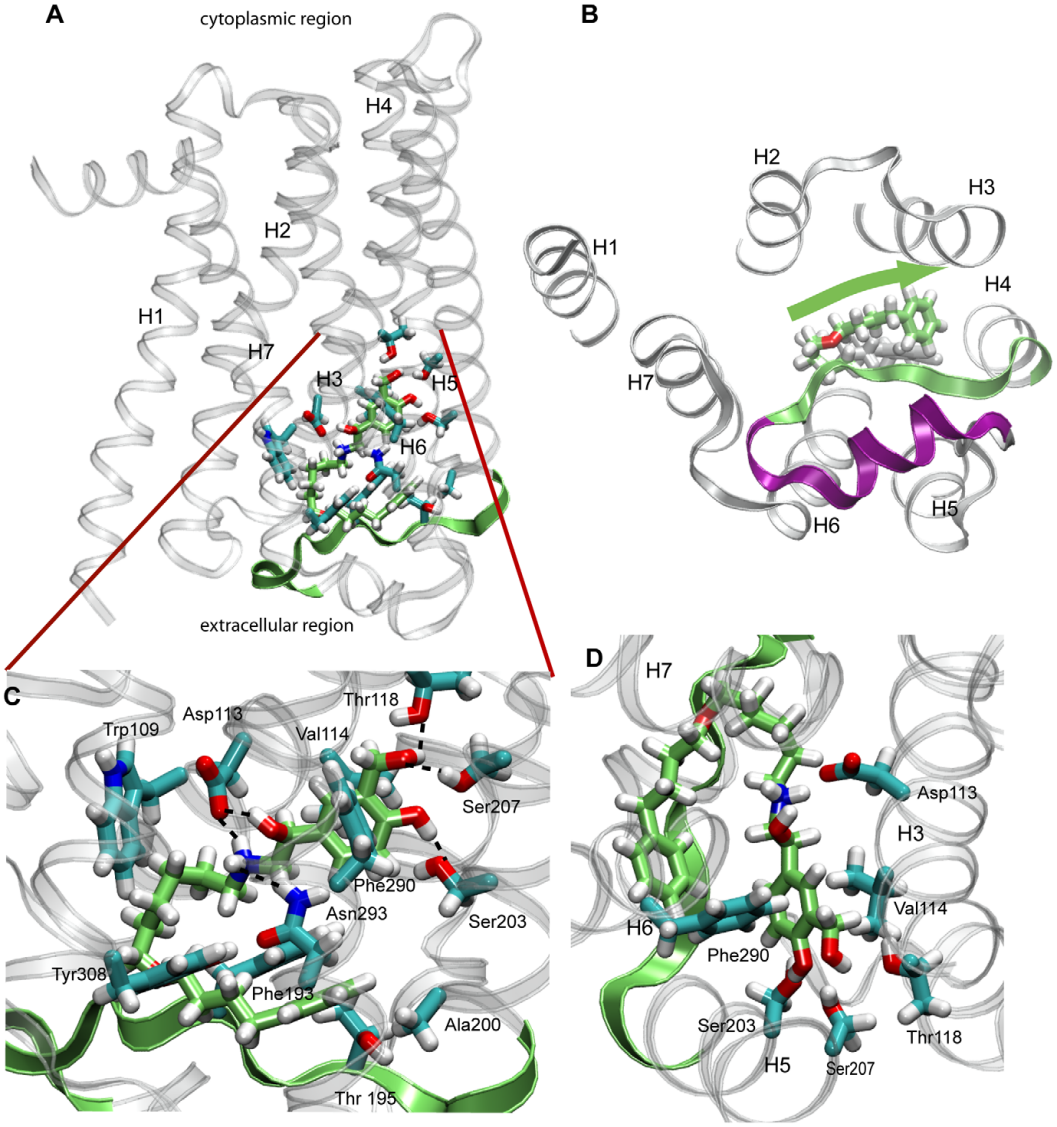
Binding of salmeterol to β_2_AR. **A.** Interactions of salmeterol at the binding site of an ANM-restrained-MD conformation; **B.** The location of salmeterol tail forming a ‘lid’ with the extracellular loop 2 (EC2) of β_2_AR from the extracellular site view. The flexible tail of salmeterol folds parallel to the EC2 and stabilizes the rest of the ligand forming a lid at the extracellular site. The green arrow shows the direction that the salmeterol tail runs similar to a beta-sheet structure. **C.** The residues lining the salmeterol binding pocket; **D.** The stabilization of the aromatic ring of salmeterol by Val114, Thr118 at H3, Ser203 and Ser207 at H5, and Phe290 atH6. Carbon, oxygen, nitrogen and hydrogen atoms of salmeterol is colored green, red, blue and white; respectively. ANM-restrained-MD conformation that is able to accommodate inside the ligand binding pocket is shown in ribbon diagram with transparent helices. The residues with the majority of atoms within the 3.5 Å making specific interactions with salmeterol are displayed. The EC2 that closely interacts with salmeterol is also show in green ribbon diagram.

Similar to a typical β_2_AR agonist, salmeterol contains a secondary amine, a chiral β-hydroxyl group, and an aromatic ring that enables it to bind to the orthosteric binding pocket. Figure 4 displays the interactions of salmeterol bound to a β_2_AR conformation from the ANM-restrained-MD. The hydroxyl groups of its aromatic ring form hydrogen bonds with Ser203 and Ser207 at H5 that are rotated toward the binding site at this conformation. These interactions are consistent with the results of mutagenesis- and molecular modeling studies showing that Ser203, Ser204, and Ser207 are the contact residues for the catechol hydroxyl groups of the agonists [33], [34], [37]. The protonated amine interacts with Asp113 at H3 and the β-hydroxyl group interacts with both Asp113 at H3 and Asn293 at H6. These interactions of the ligand serve as a bridge at the ligand binding site for connecting H3 and H6 that play a critical role for allosteric signal propagation from the ligand binding site to the cytoplasmic end of these helices to interact with the G-protein.

**Figure 5:**
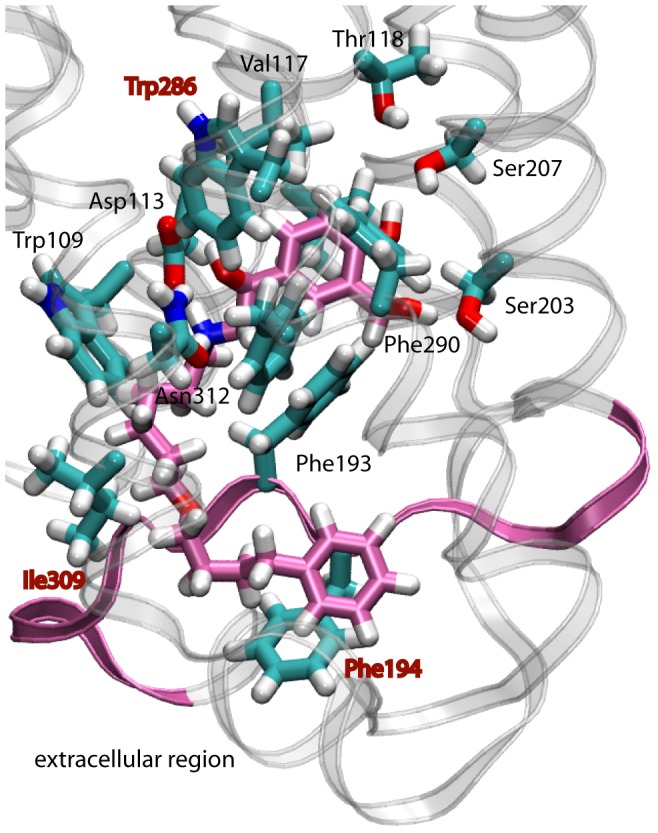
Binding of salmeterol to the active BI-167107 bound form of β_2_AR. Salmeterol occupies the same location in the crystal structure of the active β_2_AR as we identified by ANM-restrained-MD for the conformation-salmeterol complex. The residues that have atoms in 3.5 Å are displayed and those that are not interacting with salmeterol in ANM-restrained-MD conformation are labeled in red. These are Trp286 at H6, Ile 309 at H7, and Phe194 at EC2. Carbon atoms of salmeterol are colored in pink. The rest of the atoms are colored the same as in Figure 3.

Previous experiments also verified that the interactions of the protonated amine and the chiral β-hydroxyl of the agonists with Asp113 at H3 and Asn293 at H6 are necessary for β_2_AR activation [35], [36], [38]. It has also been shown that the catecholamine aromatic ring interacts with a cluster of aromatic residues (including the so-called ‘rotamer toggle switch’) that are highly conserved among the Class A GPCR family that may modulate the movement of H6, and thus may be critical for the activation of this class of GPCRs [16], [17]. Within the conformations of the β_2_AR-salmeterol complex the aromatic ring of salmeterol π-stacks with Phe290 at H6 (Fig. 4D) and with Val114 at H3, indicating that the aromatic ring is restrained between Val114 at H3 and Phe290 at H6. This finding is in agreement with the site directed mutagenesis experiments that show the size and orientation of Val114 to be critically important for the binding of aromatic rings of agonists [31]. The same interactions were observed when salmeterol was docked to the recently published active structure of β_2_AR bound to the agonist, BI-167107 [22], [41] (Fig. 5).

The docking results also show that salmeterol has a long lipophilic tail that extends to the orthosteric pocket formed by helices 3–7 (Fig. S2B). The tail region of salmeterol runs parallel to the EC2 (Fig. 4B). It has been suggested previously that salmeterol binds not only to the orthosteric pocket formed by the H3–H7 pocket of β_2_AR but also at a second site or ‘exosite’, possibly formed by a hydrophobic region of H4, or a localized membrane region in the vicinity of the receptor [42]. Docking salmeterol against the ANM-restrained-MD conformations and the crystal structure showed that the tail region of salmeterol can be accommodated at the EC region of β_2_AR by hydrophobic interactions with the extracellular site (Figs. 4, 5). The oxygen atom at the bending region of salmeterol forms a hydrogen bond interaction with Tyr308 at H7, and its aromatic ring is at the vicinity of Phe193 at EC2 (Fig. 4C). The BI-167107 bound crystal structure in the active conformation has two additional residues (Ile309 at H7 and Phe194 at EC2). These residues are labeled in red in Figure 5.

### Binding modes of the natural ligand, epinephrine

**Figure 6:**
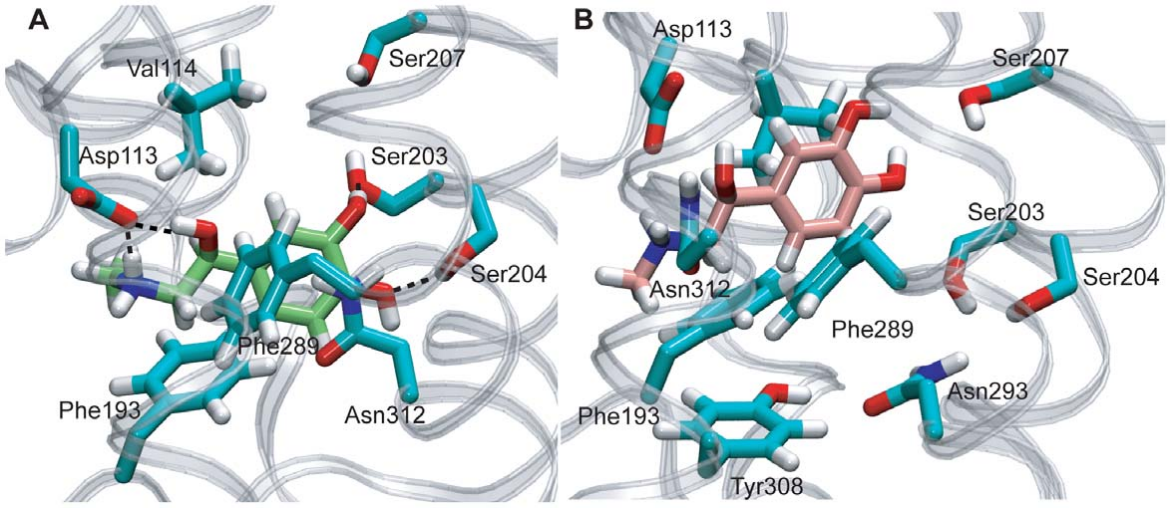
The complexes of epinephrine with an ANM-restrained-MD conformation and the active crystal structure. **A.** ANM-restrained-MD conformation-Epinephrine Complex. Epinephrine is located at the orthosteric binding site. The meta- and para- hydroxyl groups of the catechol ring are interacting with Ser203 and Ser204 at H5, respectively. Both β-hydroxyl and amine groups of epinephrine are forming hydrogen bonds with Asp113. The carbon atoms of epinephrine are colored green. **B.** BI-167107 bound active structure-Epinephrine Complex. The first pose of epinephrine in the BI-167107 bound active structure is located at the orthosteric binding pocket near the experimentally verified ligand binding residues active crystal structure. However, it is not forming any hydrogen bonds with any of these residues such as serines at H5 and Asn113 at H3.

Next, we used the ANM-restrained-MD generated conformations and the BI-167107 bound crystal structure to explore the binding modes of the endogenous ligand, epinephrine, to β_2_AR. Figure 6A shows the geometry of a complex obtained by docking epinephrine to a conformation derived from the ANM-restrained MD. In this complex, both the meta- and para-hydroxyl groups of the catechol ring of epinephrine (Fig. S2A) are interacting with Ser203 and Ser204, respectively. Furthermore, both the β hydroxyl- and the amine groups of epinephrine are forming hydrogen bonds with Asp113 in this complex. Site directed mutagenesis experiments show that interactions of both hydroxyl groups of the catechol ring with serine residues at H5 is necessary for the full efficacy of epinephrine, and that the binding affinity is abolished when either the catechol hydroxyls or the hydroxyl group on the side chain at positions of Ser203, Ser204, or Ser207 are removed [36], [37]. The amines of agonists, partial agonists, and antagonists all share an interaction with Asp113 [35]. Therefore, structural differences in the aromatic components of ligands are the primary determinants of efficacy.

Next, we analyzed the interaction of epinephrine with the BI-167107 bound active β_2_AR crystal structure (Figs. 6B, S9). The highest ranking pose of epinephrine is located at the orthosteric binding pocket near the experimentally verified ligand binding residues, but does not form hydrogen bonds with any of these residues such as serines at H5 and Asn113 at H3 (Fig. 6B). In the pose with the second highest score, epinephrine is located at the lower binding site that is suggested for binding of salbutamol closer to the EC loops [10]. At this pose, one of the hydroxyl groups of the catechol is forming a hydrogen bond interaction with Ser203, while the other hydroxyl group is closer to Tyr308 at H7 but not forming any hydrogen bond interaction. Figure S9B shows this pose from a top view from the cytoplasmic site of β_2_AR. The catechol ring of epinephrine is located between Phe193 at the EC loop 2 and Phe289 at H6, forming π-stacking interactions with both of these residues. Asp113 at H3 and Asn312 at H6 are at the closer vicinity of the β-hydroxyl and amine groups of epinephrine. Therefore, the innate ligand of β_2_AR (i.e., epinephrine) requires an alternative active β_2_AR conformer for optimal binding and does not bind the published active crystal structure of β_2_AR.

## Discussion

Understanding the conformational dynamics of β_2_AR and other GPCRs is essential to gain insights to the mechanism of action of their natural- and synthetic ligands. The studies presented here provide new insight into the dynamic behavior of GPCRs that are not addressable by static crystal structures and cover a larger part of the conformational space of the protein through the use of the ANM guided MD simulations. The extracellular site is important for ligand diffusion and stabilization and plays a critical role in the activation of β_2_AR and the duration of its active states as well as other GPCRs [1]. The motion of EC2 helix and the breakage of the salt bridge between EC2 and H7 that are predicted here might be important for both ligand diffusion and structural arrangements of the EC site in order to accommodate the ligands by coupling them to the ligand binding site. Conformational flexibility of the EC site has been also demonstrated by amide hydrogen/deuterium exchange studies [23]. Additionally, NMR data also suggested that the Asp192-Lys305 salt bridge is weakened in the β_2_AR active state and agonists induce ECs conformational changes that differ from those induced by inverse agonists [4]. Furthermore, these studies also give insights about conformational coupling between the EC site and the orthosteric binding site showing that drugs targeting these sites as allosteric modulators with high subtype selectivity.

In the present work we observed that salmeterol occupies the orthosteric binding site by forming the necessary interactions that are critical for full agonist activation (Figs. 4 and 5). The long tail of salmeterol bends and runs almost parallel to EC2 (Figs. 4 and 5). Among the notable structural differences between β_2_AR and rhodopsin are their extracellular regions: the EC2 loop of rhodopsin forms a β-strand that serves as a lid to both inverse agonist and full agonist forms of retinal, running almost parallel to its polyene chain [12], [43], [44]. Analysis of metarhodopsin II (the active state of rhodopsin) decay and folding experiments show that mutations localized near the ligand-binding pocket and EC2 are important both for the stability of both active and inactive conformations as well as for the correct folding of the protein [12], [12], [24], [45]–[47]. Furthermore, NMR data for both rhodopsin and β_2_AR showed that ligands known to differentially affect the cytoplasmic site conformation also stabilize distinct EC conformations [4]. High affinity and prolonged agonist effect of salmeterol could be explained by its additional interactions with the extracellular region of β_2_AR and forming a similar ‘lid’ for the rest of the drug that shares high structural homology with other agonists.

Salmeterol is structurally very similar to the partial agonist salbutamol except for its long and flexible tail (see Fig. S2). For the bronchodilator peak effects, 50 mg of salmeterol is equipotent to 200 mg of salbutamol and its effect lasts approximately 4 times longer than that of salbutamol. Both of these drugs have a hydroxymethyl substituent in the meta position of the aromatic ring instead of a hydroxyl group (Fig. S2) and this is the only difference between a full agonist catecholamine, such as epinephrine and salbutamol. It has been suggested that this minor difference would lead salbutamol to bind to a site at the EC region instead of the orthosteric site that catecholamines bind [10]. However, salmeterol forms the necessary interactions at the orthosteric binding pocket and the lipophilic tail region is located at the lower binding site suggested for salbutamol binding. It is possible that the stabilization of extracellular site by the tail of salmeterol would lead the rest of the molecule to diffuse to the orthosteric site and form the critical interaction for receptor activation synergistically, as has been suggested previously for other ligands [34].

We have observed that the endogenous ligand epinephrine is located at the orthosteric binding pocket forming the experimentally verified interactions in an ANM-restrained MD-epinephrine complex (Fig. 6). However, these interactions are not able to form the full agonist interactions at the orthosteric binding site of active BI-167107 bound form of β_2_AR (Fig. S9). The antagonists and inverse agonists of β_2_AR have bulky aromatic groups (see the structure of carazolol in Fig. S2) and these groups are located between the H4, H5 and H6 where agonists with smaller aromatic rings containing hydroxyl groups similar to catechol moiety of epinephrine that binds to β_2_AR. The recently determined active structure of β_2_AR has been crystallized with BI-167107. This ligand's bulky aromatic group contains the features of both agonists and antagonists and it interacts with the residues that comprise the binding pocket of both agonist and antagonists (Figs. S2 and S8) [48]. Hence the binding pocket of BI-167107 is quite large. This might be the underlying the reason for a larger agonist, such as salmeterol, forms the experimentally verified interactions with active BI-167107 bound form of β_2_AR crystal structure while epinephrine is stabilized with two aromatic residues that are close the extracellular region and it is not stabilized by the experimentally verified interactions in the orthosteric binding pocket in this structure.

While this and previous studies have shown that the members of one conserved microdomain that compromise the NPXXY motif at H7 and the Asp-Asn pair at H1 and H2, are connected through water molecules [19], [24], [49], the existence of these water molecules in the active states of β_2_AR that couple to its downstream molecules such as G-proteins still remains to be explored. The resolutions of the BI-167107 bound active states β_2_AR structures do not permit the identification of the water molecules within the protein. The binding of G-proteins leads to further conformational changes in GPCRs to stabilize their active state [8], [22], [50]. During these arrangements water molecules may be replaced by proteins coupled to GPCRs. Experimental studies have shown that the NPXXY motif at H7 and the Asp-Asn pair at H1 and H2, respectively, have additional roles in mediating interactions of GPCRs with their downstream proteins of the signaling pathway other than G-protein coupling, such as G-protein activation [32], [34], [35], [51], binding preferences to different G-proteins [14], and agonist-mediated receptor sequestration [52]. For example, the Asn391Ala mutation at the NPXXY motif of the Cholecystokinin B receptor abolishes G_q_ protein activation without affecting its association with the receptor [51]. Another site directed mutagenesis study showed that Asn376Glu mutation in the NPXXY motif within the 5-HT2A receptor changes its binding preference from small G protein to an alternative isoform [14]. It has also been shown that the mutation of Tyr326Ala completely abolishes agonist-mediated receptor sequestration of β_2_AR for undergoing rapid desensitization, and down regulating in response to agonist [52]. These findings are also verified by ^19^FMR spectroscopy showing that the NPPXY motif may be important for arrestin binding [53]. The effects of mutations on the N-D pair have also been investigated. The Glu79Asn mutation decreased the potency and maximal activity of epinephrine to stimulate cAMP accumulation generating a “constitutively inactive” mutant [35].

From a more general perspective, the interactions that a drug target has with its ligands represent a subnetwork within a network of interactions of all drugs [54], [55]. Yet, the particular protein conformations that a drug preferentially binds are ignored in these representations. However, it is a well-known fact that different states of proteins participate differentially in protein-protein interaction networks. Furthermore, binding of structurally different agonists might entail the disruption of different combinations of these intramolecular interactions, leading to different receptor conformations and differential effects on downstream signaling proteins. This information is valuable for designing functionally selective protein modulators. GPCR signaling networks are one of the best examples that illustrate the effects of conformational complexity of biomolecules in protein-protein interactions. Functional and biophysical studies show the existence of multiple, ligand-specific conformational states of GPCRs. They suggest that agonist binding and activation occur through a series of conformational intermediates linking an inactive receptor to a fully active one. These intermediate conformational states generated during agonist binding and activation may have unique functional properties and implications for cellular signaling [9], [56]. Here, we identified different conformations that form complexes with structurally different agonists (Fig. S10). Furthermore, β_2_AR is known to activate both G_s_ and G_i_, as well as non-G protein-dependent signaling pathways [5], [57], [58]. Thus, different agonists initiate specific series of signaling and regulatory events by inducing and stabilizing different sequence of conformational states. To develop a more refined understanding and abstraction of ligand- and drug-target interactions the computational approach that we have developed here as a proof of principle can be used to systematically identify functionally relevant protein conformation-drug complexes. Thus, rather than considering a protein as one single molecular species these functionally relevant conformations could be represented and viewed as individual nodes in greatly expanded protein-protein and protein-drug/ligand interaction networks (Fig. S13).

## Materials and Methods

In addition to the carazolol bound inactive crystal structure (2RH1.pdb, 2.4 Å), the initial structures of the ANM-restrained-MD simulations were derived from a previous MD study of β_2_AR [19] and kindly provided us by the Shaw group. In their study, the crystal structure of β_2_AR in its inactive conformation subjected to millisecond MD simulations using Desmond [59]. These simulations converged to two different conformations, an “open” form of the ERY motif, containing no salt bridge between Arg131 at H3 and Glu268 H6 and a “closed” form of the ERY motif. Two representative conformations was used as starting structures for ANM-restrained-MD protocol with the open form of ERY motif, one snapshot at 729 ns from the simulation of the *apo* form of β_2_AR, and the other at 515 ns from the simulations of the partial agonist, carazolol-bound form of β_2_AR. The superimpositions of these snapshots to the crystal structure of the inactive form of β_2_AR (2RH1.pdb, 2.4 Å resolution) have shown in Figure S11 and further analysis of them is provided in Text S1 page S17. The crystal structure of the inactive form of β_2_AR was also inserted into the lipids obtained from these long MD simulations. This strategy was chosen to improve on the resolution of the crystal structures and to obtain initial structures that are closer to the conformation in the membrane rather than the crystal environment.

Each system consists of β_2_AR embedded to a large, equilibrated palmitoyl-oleolyl-phosphatidyl-ethanolamine (POPE) bilayer with 170 lipid molecules, 18 sodium ions, 23 chloride ions, and 8,500 water molecules, for a total of ∼51,000 atoms, and measured 85×75×85 Å (Fig. S1A). In these systems, all Lys and Arg residues were protonated and all His, Glu, and Asp residues were deprotonated, except Glu122, which makes contacts with the lipid tails and thus likely exists in the protonated state. The system also includes the S-palmitoylation at Cys341 (shown in dark blue in Fig. S1). The procedures outlined in the Nanoscale Molecular Dynamics (NAMD) [60] tutorial on developing topology and parameter files were used to generate force field parameters for the thioester linkage between Cys341 and the palmitoyl group. There are two disulfide bridges between Cys106 and Cys191 and between Cys184 and Cys190 close to the EC site (shown in dark blue in Fig. 1C). We followed the membrane tutorial of NAMD to prepare the topology, parameter and psf files that associate these systems to run MD simulations in NAMD. To generate the ensemble of conformations using ANM-restrained-MD, we developed a fully automated code that works in NAMD and that is available from the first author (B.I.) upon request.

### The Generation of β_2_AR conformers using ANM-restrained-MD

In ANM-restrained-MD, an ensemble of ANM modes is used in an iterative scheme, as described in Figure 1B. Essentially, the algorithm generates a succession of conformations using ANM modes as harmonic restraints in MD runs, succeeded in each case by a short energy minimization algorithm to allow the molecule to settle in a local energy minimum. To this end, we first selected a pool of low frequency ANM modes. In the case of β_2_AR, the lowest frequency modes were observed to be the most cooperative ones, and the frequency distribution indicated that the subset of the first nine modes were separable. Therefore, the first nine lowest frequency modes were selected by using eigenvalue dispersion and the cooperativity criteria (see Text S1 for further details). Then, for each mode we defined two target conformations and performed short MD simulations (2500 ps) in the presence of harmonic restraints that favor these target structures. Since the restraints may lead to unrealistic strains in the structure, we also performed a short energy minimization (1,000 steps) succeeding each run and the resulting conformations were collected and used as the input conformations of the next round of ANM-restrained-MD. They are also used as the starting structures for the application of the next mode as new harmonic restraints. After screening all selected modes, this procedure ends. The underlying assumption in this protocol is that ANM-derived restraints drive the excursion of the molecule toward a direction that would otherwise be naturally selected at much longer times. A detailed description of the protocol is provided in Text S1.

### The clustering of ANM-restrained-MD generated β_2_AR conformers

For clustering of ANM-restrained-MD conformations the hierarchical clustering algorithm implemented in the XCluster [28] module of the Schroedinger 2010 Suite was utilized. First, Xcluster calculates the pairwise inter-conformational distance matrix. To define a hierarchy of clusters, it then uses an agglomerative, “single link” clustering method. It utilizes the separation ratios to determine the quality of the clusterings. The separation ratio is defined as the ratio of the shortest inter-cluster distance to the characteristic threshold distance defining the clustering. High values of the separation ratio distinguish the good clusterings. Figure S5 shows the minimum separation ratio of the clusters obtained versus the clustering level. The representative conformations that are chosen by XCluster in the clusters with the highest minimum separation ratio and the clustering level (labeled with a red circle in Fig. S5). These ligand binding residues that are listed in Figure S6D is used as the RMSD criteria of the XCluster. Clustering by the XCluster was performed using the RMSD displacements of β_2_AR residues that have been shown experimentally to be critical for the binding of drugs to β_2_AR: Trp109, Asp113, Val114 at H3; Ser203, Ser204, Ser207, and Pro211 at H5; Phe282, Trp286, Phe289, Phe290, Asn293, Ile294, and Asn297 at H6; Tyr308, Asn312, and Tyr316 at H7 [10], [16], [17], [27], [31]–[39], [61], [62]. Selection of representative conformations from each cluster gave 11, 9 and 20 conformations from the ensembles using the 729 ns Apo form, the 518 ns carazolol-bound form, and the inactive β_2_AR crystal structure, respectively. The conformations of the residues in the cluster are shown in Figure S6.

### The docking a set of known agonists of β_2_AR against the selected conformers

We first selected structurally a set of β_2_AR agonists (e.g., salmeterol, epinephrine and formoterol) with known binding affinity values for docking. In the case where three dimensional structures were available for these ligands we downloaded them from the Drugbank (http://www.drugbank.ca/). Otherwise, we built their structures using the Maestro tools of the Schrodinger suite.

Before the docking calculations we used the protein preparation wizard and grid generation tools of Schrodinger 2010 to prepare the selected β_2_ARconformers for docking. The docking studies were conducted with the Glide standard precision (SP) and extra precision XP [29], [30] module from Schrödinger for all 40 of the β_2_ARconformers.

We selected the conformations that bind the agonists using two criteria: 1. GlideXP scoring function (G-score). 2. Position and location of the agonists within the protein and their interactions with residues that are shown to be critical for the receptor binding and activation by experimental studies.

First the conformations that bind the ligands with the highest affinity have been selected by using G-score. This scoring function has been previously tested with a diverse set of ligands and receptors and it is built into the Schrodinger Suite. Briefly, G-score consist of the terms as the major potential interactions contributors to protein-ligand binding affinity such as the displacement of waters by the ligand from hydrophobic regions of the protein active site, protein–ligand hydrogen-bonding as well as other strong electrostatic interactions, desolvation effects, entropic effects due to the restriction on binding of the motion of flexible protein or ligand groups. The pose viewer tool of the Glide XP displays the different conformations/poses of ligands within the complexes in the order of their corresponding G-scores. Each ANM-restrained-MD conformation and an agonist complex generated by Glide XP has been analyzed using the pose viewer tool and the agonist pose with the highest G-scores have been chosen for each protein conformation.

Second, we collected the ligand and protein interactions that are crucial for the ligand binding and the protein activation from the literature. For example, it has been shown that the agonists with the catecholamine moiety interact with at least two of the three Serine residues (Ser203, Ser204 and Ser207) at H5. It is also well demonstrated that the interaction of Asp113 at H3 with certain ligands with their hydroxyl and amines groups) are crucial for stabilizing them in the binding pocket. The amino acids that are involved in the ligand binding and activation have been identified by site-directed mutagenesis, fluorescence spectroscopy, constitutively active and inactive mutants of β_2_AR as well as the different derivative of the agonists. Table 1 lists these residues and the agonist motives that bind to them along with the studies that identified the interactions [10], [16], [17], [27], [31]–[39], [61], [62].

### The docking of a compound derived from a different crystal structure

The aim of the protocol that was described above was to find conformations that accommodate agonists with different structures and sizes by starting with an inactive structure that cannot reproduce the interactions that are known to be critical for the agonist binding and protein activation. Additionally, we tested this protocol by using a compound as a probe of conformations derived from a different crystal structure. To this end, we retrieved an inactive crystal structure of alprenolol-β_2_AR complex (3NYA.pdb and 3.16 Å resolution) from the Protein Data Bank. Subsequently, we docked alprenolol to the ANM-restrained-MD generated conformations. Then we screened the conformation-ligand complexes having the highest binding G-scores for the interactions that have formed in the crystal structure. The complex that reproduced these interactions and has the closest side chain and the ligand conformations is displayed in Figure S12A and Figure S12B displays the interactions of alprenolol in the crystal structure.

## Supporting Information

Text S1 This supporting information provide further details for: **A.** Generating protein conformers with ANM-restrained MD **B.** Comparison of the snapshots from the millisecond scale MD simulations of tthe inactive β_2_AR that are obtained from the Shaw group with the crystal structure of the inactive β_2_AR with carazolol **C.** Binding of Alperenolol to ANM-restrained-MD conformations.
(PDF)

Figure S1 **The β_2_AR system and the schematic view for generating the tree of the conformation ensemble by ANM-restrained MD protocol A.** β_2_AR embedded into a lipid bilayer and hydrated by water molecules. The group that attaches Cys341 to the membrane as well as the two disulfide bridges between Cys106 and Cys191 and between Cys184 and Cys190 are colored in blue. **B.** The schematic view for generating the tree of the conformation ensemble by ANM-restrained-MD protocol using three modes. For illustration purposes the ribbon diagrams of the starting- and two conformations (in opposite directions, plus and minus,) derived from the first mode of adenylate kinase are shown.
(TIF)

Figure S2 **The ligands of β2AR that are used in this study.**
(TIF)

Figure S3 **Root mean square per time profile of ANM-restrained MD simulations of β_2_AR.** Figure 3A and 3B illustrate the representative time evolution of the RMSD in C^α^ positions from the initial structures. Figure 3A displays the RMSDs for the ten slowest modes that are used as targets in the ANM-restrained-MD simulations. The two curves in each graph refer to the opposite direction deformations. By the end of each simulation the conformation departs from the original one by an RMSD of about 1.5 Å. The modes that are used as targets are labeled for each graph are labeled. Blue and red curves represent the simulations in plus and minus directions, respectively. Figure 3B shows a representative time evolution of RMSD in C^α^ positions from the initial structure in the ANM-restrained-MD simulations iteratively, as described in the Methods section.
(TIF)

Figure S4 **Ribbon diagrams of β_2_AR ANM-restrained-MD conformations.** Front view (A-left column) and back view (B-left column) of β_2_AR conformers generated by the ANM-restrained-MD are shown. Cell membrane-spanning helices and extracellular and cytoplasmic regions are shown in different colors and denoted by numbers. Water molecules stabilizing the conserved NPXXY motif (A-right column) and the Asn-Asp pair within the transmembrane region and the critical catecholamine binding Ser203 and Ser207 residues at H5 (B-right column) in conformations where they both point to the ligand binding pocket are shown.
(TIF)

Figure S5 **Clustering ANM-restrained-MD β_2_AR conformations.** Clustering results of β_2_AR conformations by the XCluster module of Schrodinger. The residues that are shown to play a critical role in the binding of ligands to β_2_AR were used as root mean square fluctuations criteria of XCluster. The clusters with the highest minimum separation ratio and the clustering level have been selected.
(TIF)

Figure S6 **XCluster-selected conformations of β_2_AR.** The side chains of the binding site residues that are used in the clustering criteria in XCluster are shown at H3 and H4 (A), H5 and H6 (B) and the extracellular region (C). The ligand binding residues that are used in XCluster as RMSD criteria are listed (D).
(TIF)

Figure S7 **The comparison of the target ANM modes and their corresponding ANM-restrained-MD conformations.** The left panels show the RMSD per residue profiles between the ANM modes and their corresponding ANM-restrained MD conformations for mode 8 in minus direction (8M) that forms the complex with salmeterol (A) and for mode 2 in plus direction (2P) that forms the complex with epinephrine. The right panels show ribbon diagrams of 8M (A) and 2P (B) β_2_AR conformations. The CA carbons of ANM modes and their corresponding ANM-restrained MD conformations are diplayed as red and blue spheres respectively. The helices are colored in accord with the rest of the manuscript. The color code of the helices that are used in the ribbon diagrams of β_2_AR are also displayed as colored bands at the bottom of the graphs.
(TIF)

Figure S8 **The size and the location of β_2_AR agonists in the protein-ligand complexes.** The head groups of all agonist are located at the orthosteric binding pocket and they interact with the Serine residues at H5 rotated towards the binding pocket. While epinephrine populates only the orthosteric binding site both BI-167107 and salmeterol protrude to the extracellular region. The head groups of all agonist are located at the orthosteric binding pocket interacting with the Serine residues at H5 rotated towards the binding pocket. Ribbon diagrams of β_2_AR conformations are shown in gray. Molecular surfaces of the ligands are created by using MOE. Green, purple and blue regions of the ligands are hydrophobic, hydrophilic and mildly polar, respectively.
(TIF)

Figure S9 **Binding of epinephrine to the active BI-167107 bound form of β_2_AR.** **A.** The first pose of epinephrine in the BI-167107 bound active structure is located at the orthosteric binding pocket near the experimentally verified ligand binding residues active crystal structure. However, it is not forming any hydrogen bonds with any of these residues such as serines at H5 and Asn113 at H3. **B.** In the second highest rank pose of epinephrine in the active BI-167107 bound structure it is located at the lower binding site closer to the EC site. At this pose, one of the hydroxyl groups of the catechol is forming a hydrogen bond interaction with Ser203 while the other hydroxyl group is closer to Tyr308 at H7 but not forming any hydrogen bond interaction. Figure S9B shows this pose from a top view from the cytoplasmic site of β_2_AR. The catechol ring of epinephrine is located between Phe193 at the EC loop 2 and Phe289 at H6 forming π-stacking interactions with both of these residues. Asp113 at H3 and Asn312 at H6 are at the closer vicinity of the β-hydroxyl and amine groups of epinephrine. The epinephrine molecules in complex with the active BI-167107 bound structure are shown in pink color.
(TIF)

Figure S10 **Binding of modes salmeterol, R, R formoterol and epinephrine to β_2_AR.** ANM-restrained MD conformations that accommodate β_2_AR agonists with experimentally verified interactions are shown. The ANM-restrained MD conformations-ligands complexes are with salmeterol, R, R formoterol and epinephrine are shown in the left-, center- and right panels, respectively. The side chains of the residues that are 3.5 Å of the ligands are displayed. Coloring of the atoms are the same as in Figure 4. The EC2 that interacts with salmeterol is shown in green in the first panel. All β-hydroxyl and protonated amine groups of the ligands are forming hydrogen bonds with Asp113 at H3. The hydroxyl groups of the aromatic rings of all ligands are forming hydrogen bonds with Serines at H5. Epinephrine binds to the orthosteric binding site slightly closer to the EC site compared to the larger ligands, salmeterol and R, R formoterol.
(TIF)

Figure S11 **The Comparison of the millisecond scale MD simulation snapshots with the inactive crystal structure of β_2_AR.** The left panels show the ribbon diagrams of the apo (A) and carazolol (B) bound snapshots that are superimposed to the inactive carazolol bound crystal structure. The most pronounced conformational changes are denoted by the arrows. The right panels show the corresponding binding sites of these snapshots compared to the crystal structure. The carbon atoms of the MD snapshots and the crystal structure side chains are colored cyan and green, respectively. The rest of the atoms and the helices are colored in accord with the rest of the document.
(TIF)

Figure S12 **Alprenolol in ANM restrained MD conformation and in the crystal structure complex.** Binding residues of alprenolol in ANM-restrained-MD conformation (A) and in the crystal structure (B). Carbon atoms of alprenolol in ANM-restrained-MD conformation (6P) and the crystal structures colored green and pink, respectively. The heavy atoms of the residues that are in the 4.5 A of alprenolol are displayed and labeled. Both structures have the same residues in the vicinity of alprenolol and display similar orientations. Unlike agonists of β_2_AR alprenolol does not contain hydroxyl groups that interact with the Serine residues at H5. In both structures Ser203 and Ser207 are not rotated towards the binding cavity of β_2_AR. Instead, the head group of alprenolol is stabilized at the binding site with the π-stacking interaction with the so-called rotamer toggle switch, Phe290 at H6. The rest of the atoms are colored in the same pattern as in the rest of the manuscript.
(TIF)

Figure S13 **Refining drug target network by assessing protein motion.** In current drug target network representations drug targets (gray circles) are interpreted as single entities connected through drugs (black circles), and the particular biologically relevant protein conformations that a drug preferentially binds are ignored. However, to fully understand the interactions of drugs to their targets a target should be represented by its different functionally relevant conformations (different colored shapes within grey line enclosed areas). Drug targets that are represented by single structures are connected to drugs by blue dashed lines. The target conformations that preferentially bind a drug are connected by red dashed lines.
(TIF)
