# Supplementary material for: Identifying Ligand Binding Conformations of the β2-Adrenergic Receptor by Using Its Agonists as Computational Probes
Source: PLoS One. 2012 Dec 31;7(12):e50186. doi: 10.1371/journal.pone.0050186 (PMC3534076; doi:10.1371/journal.pone.0050186)
Supplement: Text S1 — This supporting information provide further details for: A. Generating protein conformers with ANM-restrained MD B. Comparison of the snapshots from the millisecond scale MD simulations of tthe inactive β2AR that are obtained from the Shaw group with the crystal structure of the inactive β2AR with carazolol C. Binding of Alperenolol to ANM-restrained-MD conformations. (PDF) [file pone.0050186.s001.pdf]

Identifying ligand binding conformations of the β2-adrenergic receptor by using its agonists as computational probes

Supporting Information

Basak Isin[1], Guillermina Estiu[2], Olaf Wiest[2,3] and Zoltán N. Oltvai[1]

[1]Department of Pathology, University of Pittsburgh, Pittsburgh, PA, 15261, USA
[2]Department of Chemistry and Biochemistry, University of Notre Dame, Notre Dame, IN, 46556
[3]School of Chemical Biology and Biotechnology, Peking University, Shenzhen Graduate School, Shenzhen, 518055, China

Contents

A. Generating protein conformers with ANM-restrained-MD pS2-4

B. The comparison of the snapshots from the millisecond scale MD simulations of the inactive β_2AR with the crystal structure of the inactive β_2AR with carazolol pS5

C. Binding of Alperenolol to ANM-restrained-MD conformations pS6

References pS6

Supplementary Figures List

Figure S1 The β_2AR system and the schematic view for generating the tree of the conformation ensemble by ANM-restrained-MD protocol

Figure S2 The ligands of β_2AR that are used in this study

Figure S3A Root mean square per time profile of ANM-restrained-MD simulations of β_2AR

Figure S3B Root mean square per time profile of ANM-restrained-MD simulations of β_2AR

Figure S4 Ribbon diagrams of β_2AR conformations

Figure S**5** Clustering the ANM-restrained-MD β_2AR conformations

Figure S6 XCluster-selected conformations of β_2AR

Figure S7 The comparison of the target ANM modes and ANM-restrained-MD conformations

Figure S8 The size and the location of β_2AR agonists in the protein-ligand complexes

Figure S9 Binding of epinephrine to the active BI-167107 bound form of β_2AR

Figure S10 Binding of modes salmeterol, R, R formoterol and epinephrine to β_2AR

Figure S11 The comparison of millisecond scale MD snapshots with crystal structure of β_2AR

Figure S12 Alprenolol in ANM restrained MD conformation s

Figure S13 Refining drug target network by assessing protein motion

A. Generating protein conformers with ANM-restrained MD

ANM-restrained MD protocol

1. Generating the normal modes: ANM is utilized for generating the normal modes. ANM, described in detail previously [1], is a normal mode analysis that uses as potential

$$V_{ANM} = \frac{\gamma}{2}\sum_{i=1} (|R_{ij}| - |R_{ij}^{0}|)^2 h(R_{cut}^{ANM} - |R_{ij}^{0}|) \qquad (1)$$

where [illegible] and [illegible] denote the original and instantaneous distance vectors between residues *i* and *j*, represented by their C^{α}-atom positions, *h(x)* is the Heavyside step function equal to 1 if x is positive, and zero otherwise, R_{cut}^{ANM} is the cutoff distance for inter-residue interactions, taken as 13 Å [2], and γ is the force constant of the order of 1 kcal/(mol Å^2), usually found by requiring the sum of $<(\Delta \mathbf{R}_i)^2>$ over all residues ($1 \leq i \leq N$) to match the sum of the experimental B-factors. The mode shapes are insensitive to the absolute value of γ. The cross-correlation $<\Delta \mathbf{R}_i . \Delta \mathbf{R}_j>$ between the fluctuations of residues *i* and *j* can be expressed as a sum

$$<\Delta \mathbf{R}_i . \Delta \mathbf{R}_j> = \Sigma_k [\Delta \mathbf{R}_i . \Delta \mathbf{R}_j]_k = \sum_k (3 k_B T / \gamma) \left[\lambda_k^{-1} \mathbf{u}_k \mathbf{u}_k^T\right]_{ij} \qquad (2)$$

over the contributions of all modes (*k*), using the eigenvectors $\mathbf{u}_k$ and eigenvalues λ_k of the Hessian **H**, evaluated from the second derivatives of V_{ANM}. Here k_B is the Boltzmann constant, *T* is the absolute temperature, and a total of $1 \leq k \leq 3N-6$ nonzero modes contribute to $<\Delta \mathbf{R}_i . \Delta \mathbf{R}_j>$. $\mathbf{u}_k$ describes the normalized mobility profile of residues induced by mode *k*, $\lambda_k^{1/2}$ scales with its frequency.

2. Selection of Distinctive and Cooperative Modes: The lowest frequency modes of ANM were chosen as they represent the functional motions[3]. To further determine the most relevant lowest frequency modes two criteria were considered: mode frequency dispersion (or eigenvalue distribution) and the degree of collectivity. The mode frequency dispersion is examined to identify a subset that has distinctive frequencies. The degree of collectivity [4], on the other hand, is calculated using

$$\kappa = \frac{1}{N}\exp(-\sum^{N} \alpha \Delta \mathbf{R}_i^2 \log \alpha \Delta \mathbf{R}_i^2) \qquad (3)$$

where α is a normalization factor to obtain $\sum^{N} \alpha \Delta \mathbf{R}_i^2 = 1$ [3] to ascertain that the selected modes are cooperative enough. This criterion is useful for eliminating the cases where the low frequency modes induce a motion in a loosely coupled chain segment only (e.g., the *N*- or *C*-terminus).

3. Preparing the target conformations: Since each mode corresponds to a fluctuation between two oppositely directed motions, both directions being equally probable, we considered two sets of deformations for each mode, referred to as 'plus' or 'minus' displacements along the particular mode axis. The corresponding 'target' conformations require the root-mean-square displacements (RMSD) in C^{α}-atom positions to remain close to 1.5 Å after reconfiguration along mode 1, and $1.5(\lambda_i/\lambda_1)^{1/2}$ Å for mode *i*.

4. Applying harmonic restraints along the ANM modes in MD: Each C^{α} atom is harmonically restrained to approach the target conformations for 20 picoseconds. The instantaneous distance $\rho(t)$ of each configuration from the target configuration can be written as:

$$\rho(t) = |\mathbf{r}(t) - \mathbf{r}_T| = \left(\sum (\mathbf{r}_i(t) - \mathbf{r}_{Ti})^2\right)^{\frac{1}{2}} \quad (4)$$

where $\mathbf{r}(t)$ is the conformation vector of the biomolecule and $\mathbf{r}_i(t)$ is the position vector of the atom *i* at time *t*. Likewise, $\mathbf{r}_T$ is the target conformation vector for the biomolecule and $\mathbf{r}_{Ti}$ is the position vector of the atom *i* at the target conformation.

The following restraint is used to reach the target structure:

$$\Phi(\mathbf{r}(t)) = \sum (\mathbf{r}_i(t) - \mathbf{r}_{Ti})^2 - \rho^2(t) = 0 \quad (5)$$

The additional force in MD due to the implementation of this constraint can be written as:

$$\mathbf{F}_c(t) = k\frac{\partial \Phi}{\partial t} = 2k(\mathbf{r}(t) - \mathbf{r}_T) \quad (6)$$

where *k* is the force constant.

A typical MD simulation with a harmonic restrains consists of the following steps as described in [5]

a. Set the distance ρ_I between target and initial conformation ($\mathbf{r}_I$) as $\rho_I = |\mathbf{r}_I - \mathbf{r}_T|$

b. Solve the equation of motion containing the additional $\mathbf{F}_c$ for the constraint, by assigning initial coordinates using the initial conformation and an appropriate set of initial velocities.

c. At each succeeding timestep, decrease the distance by $\Delta\rho = (\rho_I - \rho_T)\frac{\Delta t}{t_s}$ where t_s is the simulation time and ρ_T, is the distance of the conformation from the target structure at the end of the simulation, which should be as small as possible. The distance $\rho(t)$ from the target structure is gradually decreased during the course of simulation.

5. Energy Minimization: After reaching the two target structures for a given mode, both are subjected to 1,000 steps of energy minimization using the steepest descent algorithm to relieve possible unrealistic distortions.

B. Comparison of the snapshots from the millisecond scale MD simulations of the inactive β_2AR with the crystal structure of the inactive β_2AR with carazolol

In addition to the inactive carazolol-bound crystal structure of β_2AR, two snapshots that were derived from a previous millisecond scale MD study of β_2AR were used as the initial structures for the ANM-restrained-MD simulations. As stated in the original manuscript, these micro- to millisecond scale MD simulations have explored the inactive state properties of the β_2AR. The superimpositions of the snapshots taken from the MD simulations to the starting inactive crystal structure are shown in Figure S11. The left panels display the ribbon diagrams snapshots from the MD simulations of the apo (Fig 11A) and carazolol bound structures (Fig 11B), superimposed to the crystal structure. Both of the snapshots contain no significant backbone movements, precluding the structural rearrangements that are crucial for agonist binding and activation. However, in the apo structure the side chains of the amino acids that line the binding site went through larger structural rearrangements due to the absence of a ligand at the cavity. The highly conserved Asp 113 in H3 that forms a hydrogen bond with β-OH motif of the agonists had a displacement of 3.95 Å from the initial crystal structure. Additionally, aromatic residues Phe193 at the EC2 and Tyr308 at H7 show displacements of 5.95 Å and 5.35 Å, respectively (Figure S11). Due to these rearrangements in the absence of a ligand, both the apo structure and the conformations that are obtained by the ANM-restrained-MD simulations of this structure did not form any complexes with the agonists that have experimentally verified interactions. In contrast to the apo structure, the snapshot from the MD simulation of the carazolol bound, inactive β_2AR had no substantial conformational changes at the side chains of the binding site residues (Figure S11 B right panel). The residues Phe193 at the EC2 and Tyr308 at H7 again show the largest displacements (0.71Å and 0.63 Å, respectively) and Asp 113 at H3 has no conformational change at this snapshot.

C. Binding of Alperenolol to ANM-restrained-MD conformation

In addition to docking of salmeterol and epinephrine, we retrieved an inactive crystal structure of alprenolol-β_2AR complex (3NYA.pdb) from the Protein Data Bank. Subsequently, we docked alprenolol to the ANM-restrained-MD generated conformations. We performed virtual screening for the conformations of the GPCR-ligand complexes having the highest binding G-scores for the interactions that are observed in the crystal structure. The complex that reproduced these interactions and has the closest side chain and the ligand conformations is displayed in Figure S12A. In turn, Figure S12B shows the interactions of alprenolol in the crystal structure. The heavy atoms of the residues that are within 4.5 Ǻ of alprenolol are displayed and labeled. Carbon atoms of alprenolol in ANM-restrained-MD conformation (the sixth mode along the positive direction, 6P) and the crystal structures are colored green and pink, respectively. The rest of the atoms are colored in the same pattern with the rest of the manuscript. Both structures have the same residues in the vicinity of alprenolol and display similar orientations. As an antagonist, alprenolol does not contain hydroxyl groups its aromatic head group to interact with the Serine residues at H5. Therefore, Ser203 and 207 at H5 are not rotated towards the binding cavity of β_2AR as observed in the crystal structure and the ANM-restrained-MD conformation. Instead, the head group of alprenolol is stabilized at the binding site with the π-stacking interaction with the so-called rotamer toggle switch residue Phe290.

Reference

1. Atilgan AR, Durell SR, Jernigan RL, Demirel MC, Keskin O, et al. (2001) Anisotropy of fluctuation dynamics of proteins with an elastic network model. Biophys J 80: 505-515.

2. Eyal E, Chennubhotla C, Yang LW, Bahar I (2007) Anisotropic Fluctuations of Amino Acids in Protein Structures: Insights from X-Ray Crystallography and Elastic Network Models. Bioinformatics in press.

3. Tama F, Sanejouand YH (2001) Conformational change of proteins arising from normal mode calculations. Protein Eng 14: 1-6.

4. Bruschweiler R (1995) Collective Protein Dynamics and Nuclear-Spin Relaxation. Journal of Chemical Physics 102: 3396-3403.

5. Schlitter J, Engels M, Kruger P (1994) Targeted molecular dynamics: a new approach for searching pathways of conformational transitions. J Mol Graph 12: 84-89.
