# Supplementary figures and images for: Identifying Ligand Binding Conformations of the β2-Adrenergic Receptor by Using Its Agonists as Computational Probes
Source: PLoS One. 2012 Dec 31;7(12):e50186. doi: 10.1371/journal.pone.0050186 (PMC3534076; doi:10.1371/journal.pone.0050186)

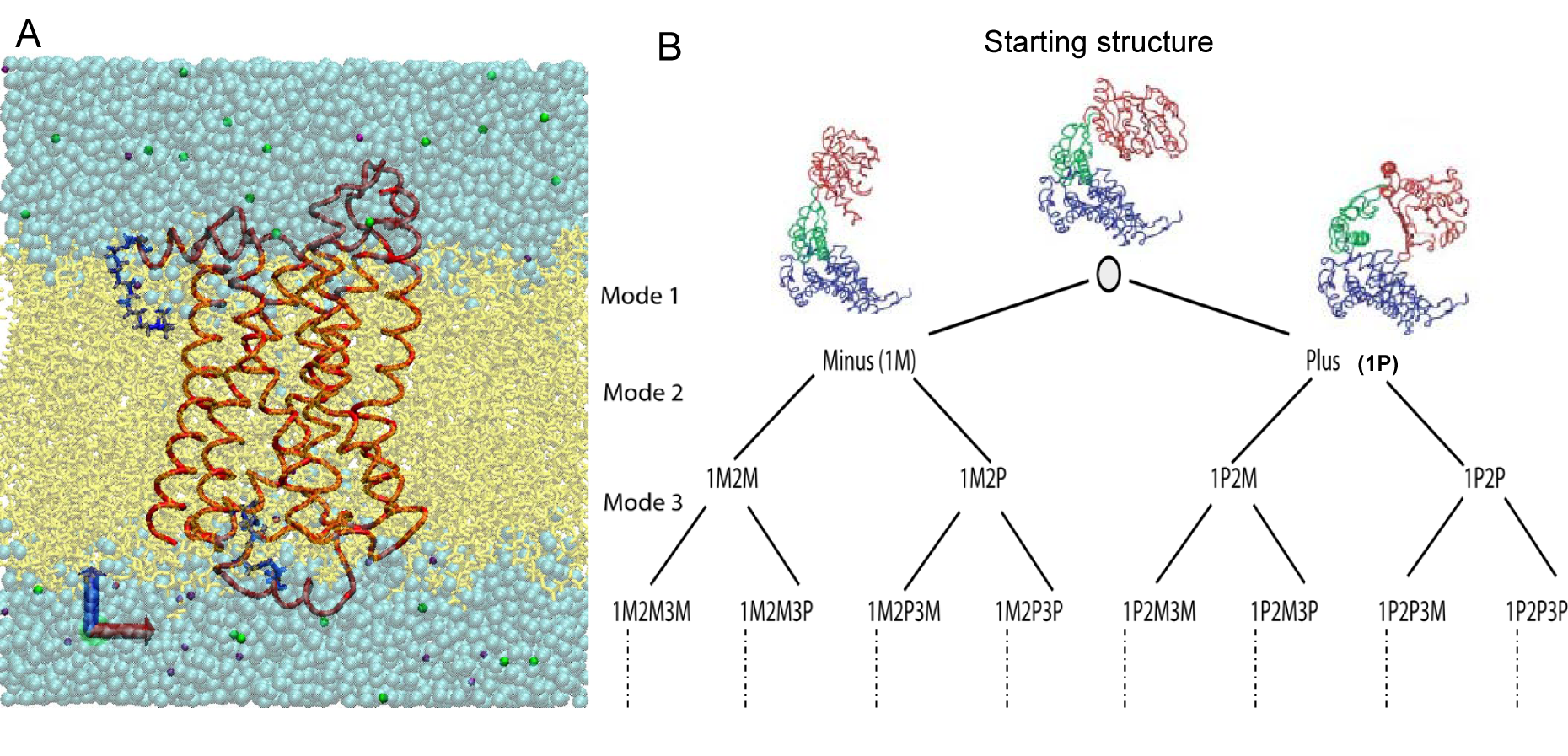

Supplement: Figure S1 — The β2AR system and the schematic view for generating the tree of the conformation ensemble by ANM-restrained MD protocol A. β2AR embedded into a lipid bilayer and hydrated by water molecules. The group that attaches Cys341 to the membrane as well as the two disulfide bridges between Cys106 and Cys191 and between Cys184 and Cys190 are colored in blue. B. The schematic view for generating the tree of the conformation ensemble by ANM-restrained-MD protocol using three modes. For illustration purposes the ribbon diagrams of the starting- and two conformations (in opposite directions, plus and minus,) derived from the first mode of adenylate kinase are shown. (TIF) [file pone.0050186.s002.tif]

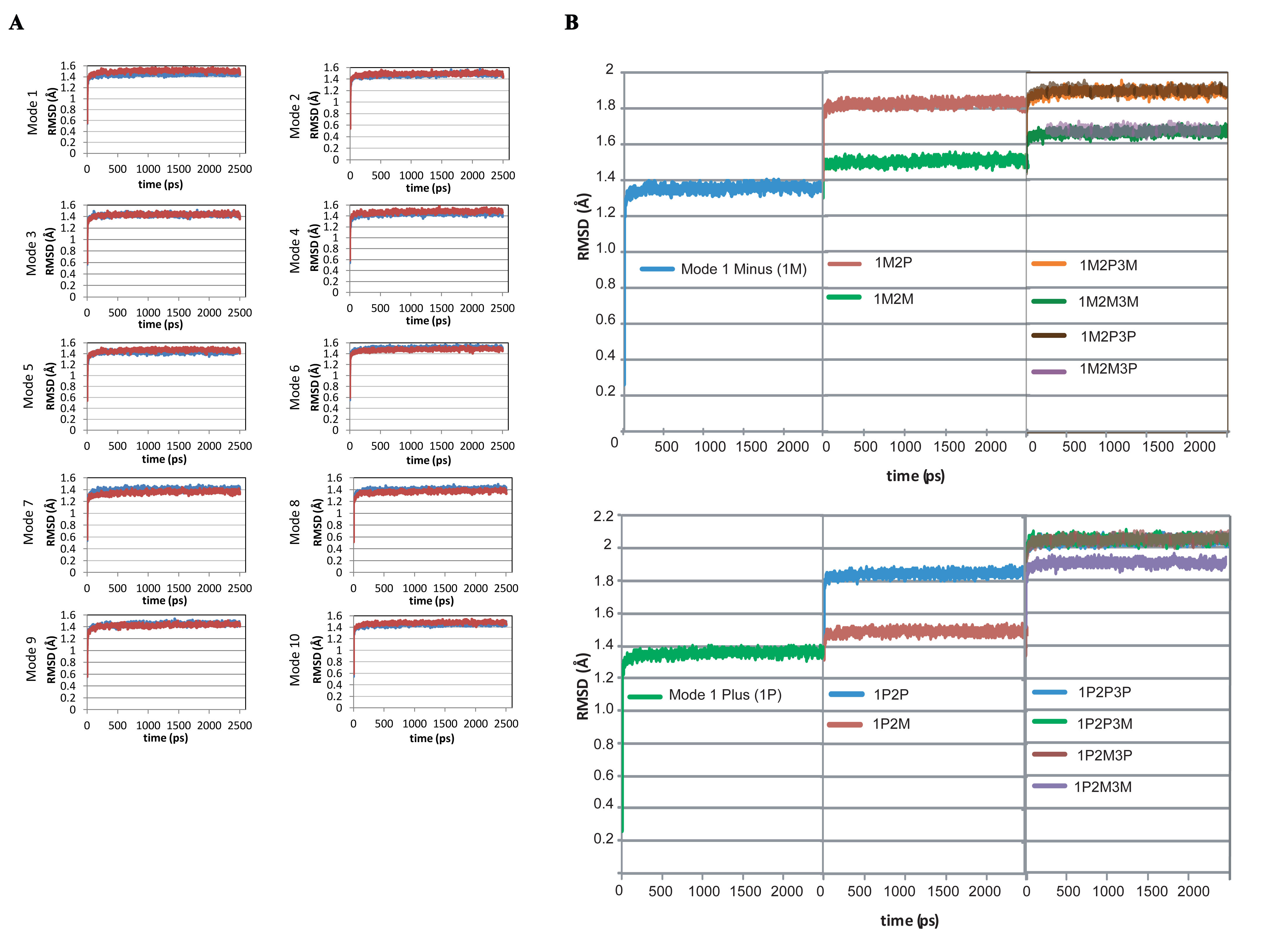

Supplement: Figure S3 — Root mean square per time profile of ANM-restrained MD simulations of β2AR. Figure 3A and 3B illustrate the representative time evolution of the RMSD in Cα positions from the initial structures. Figure 3A displays the RMSDs for the ten slowest modes that are used as targets in the ANM-restrained-MD simulations. The two curves in each graph refer to the opposite direction deformations. By the end of each simulation the conformation departs from the original one by an RMSD of about 1.5 Å. The modes that are used as targets are labeled for each graph are labeled. Blue and red curves represent the simulations in plus and minus directions, respectively. Figure 3B shows a representative time evolution of RMSD in Cα positions from the initial structure in the ANM-restrained-MD simulations iteratively, as described in the Methods section. (TIF) [file pone.0050186.s004.tif]

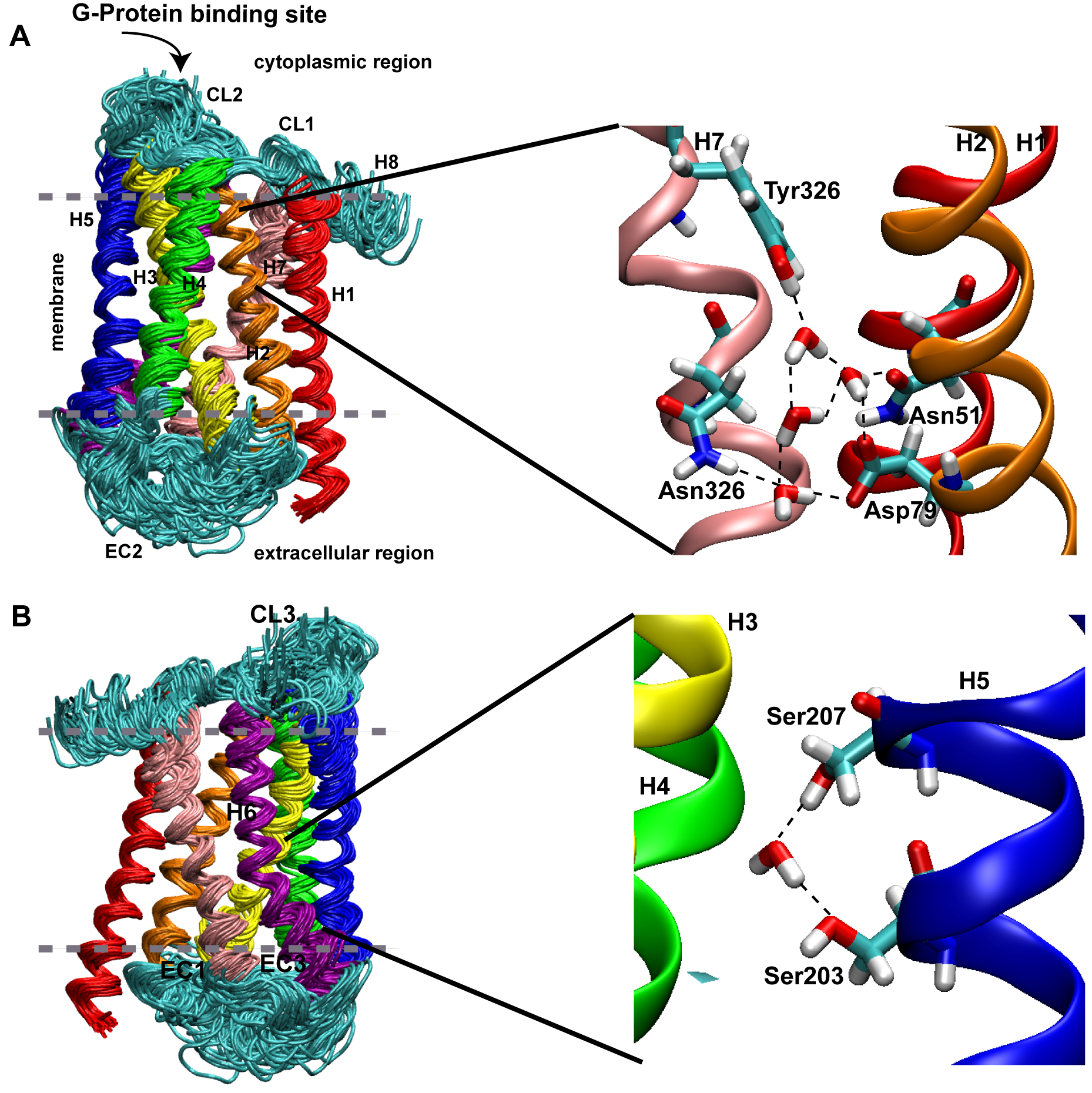

Supplement: Figure S4 — Ribbon diagrams of β2AR ANM-restrained-MD conformations. Front view (A-left column) and back view (B-left column) of β2AR conformers generated by the ANM-restrained-MD are shown. Cell membrane-spanning helices and extracellular and cytoplasmic regions are shown in different colors and denoted by numbers. Water molecules stabilizing the conserved NPXXY motif (A-right column) and the Asn-Asp pair within the transmembrane region and the critical catecholamine binding Ser203 and Ser207 residues at H5 (B-right column) in conformations where they both point to the ligand binding pocket are shown. (TIF) [file pone.0050186.s005.tif]

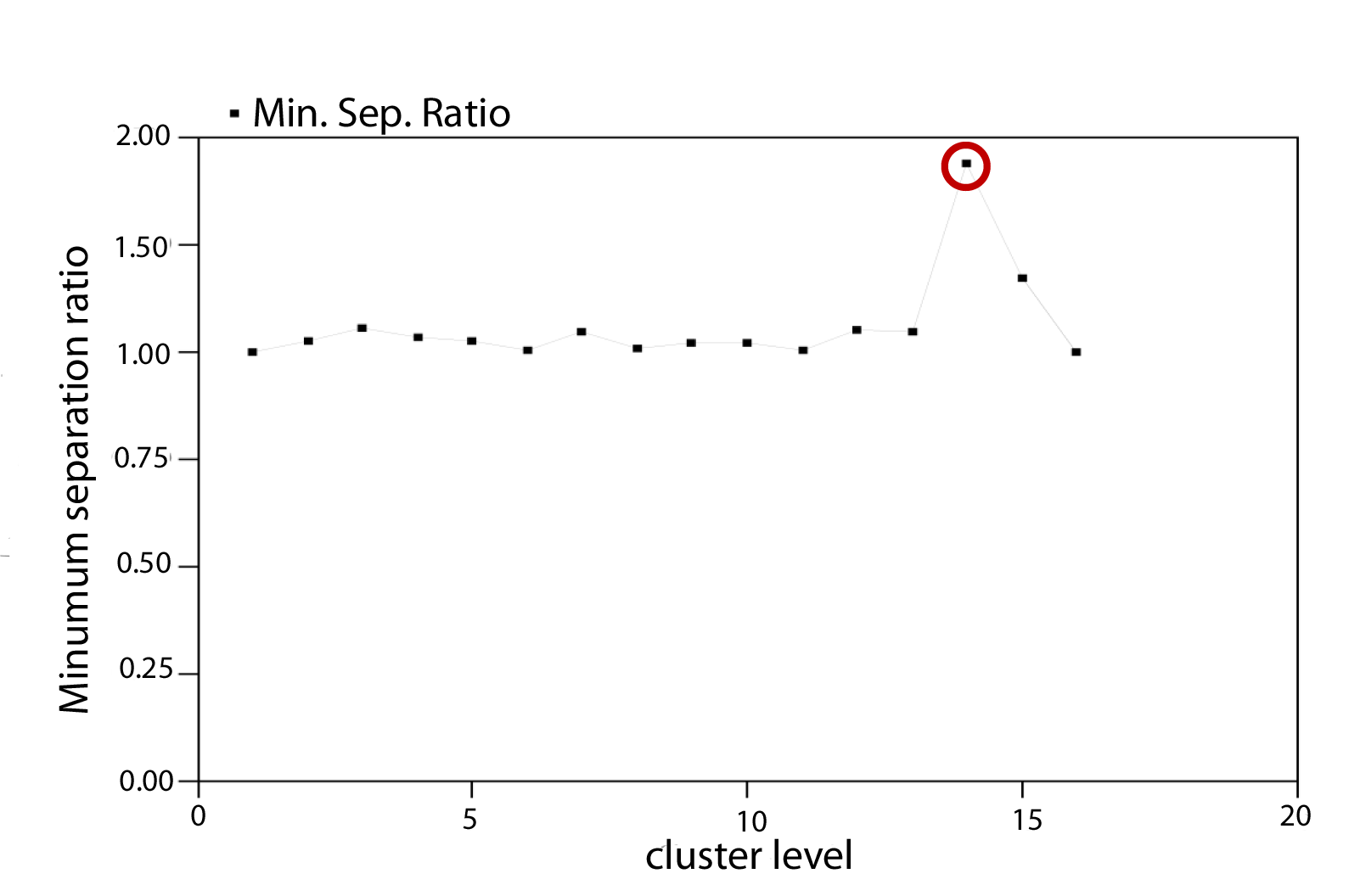

Supplement: Figure S5 — Clustering ANM-restrained-MD β2AR conformations. Clustering results of β2AR conformations by the XCluster module of Schrodinger. The residues that are shown to play a critical role in the binding of ligands to β2AR were used as root mean square fluctuations criteria of XCluster. The clusters with the highest minimum separation ratio and the clustering level have been selected. (TIF) [file pone.0050186.s006.tif]

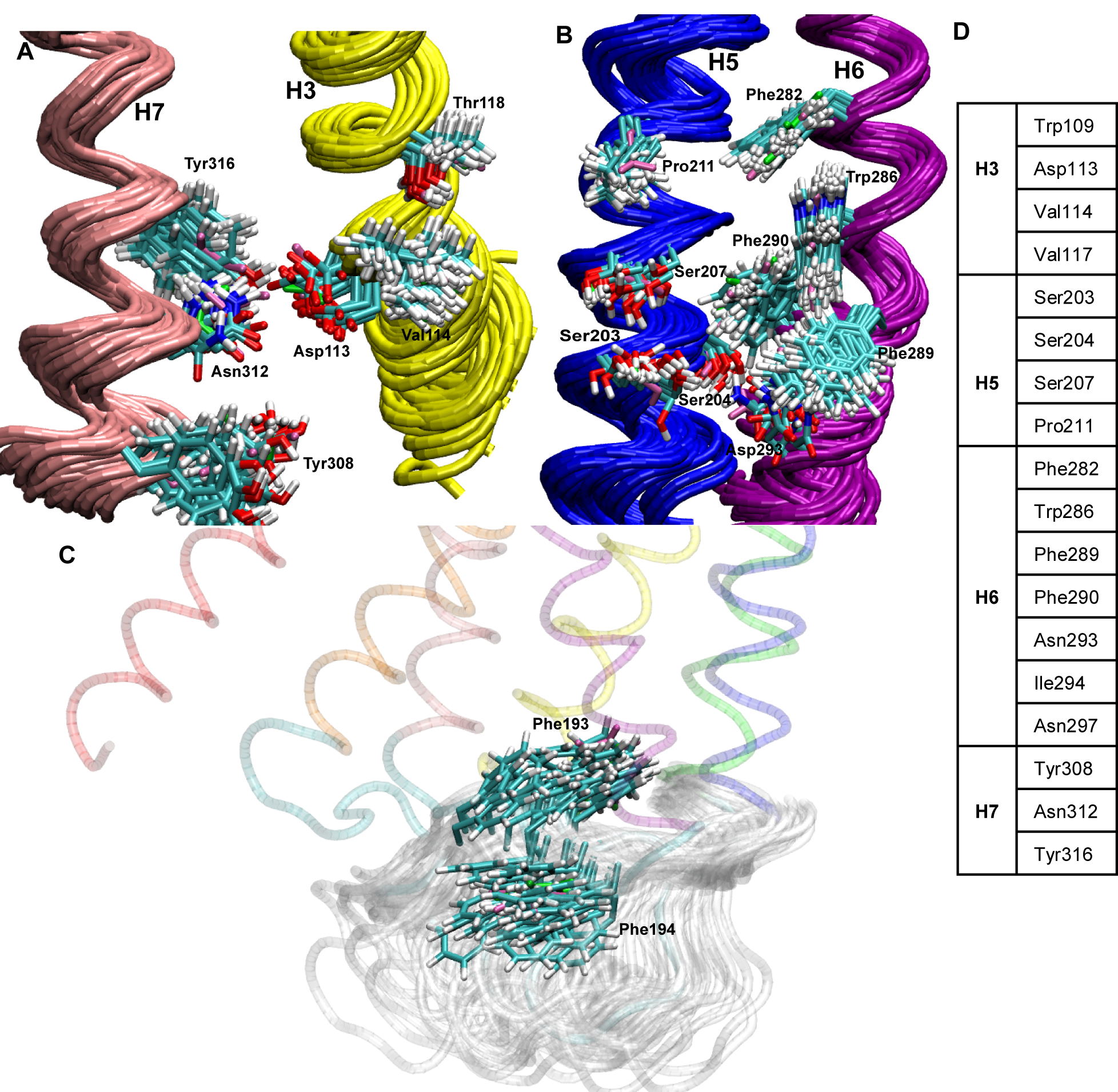

Supplement: Figure S6 — XCluster-selected conformations of β2AR. The side chains of the binding site residues that are used in the clustering criteria in XCluster are shown at H3 and H4 (A), H5 and H6 (B) and the extracellular region (C). The ligand binding residues that are used in XCluster as RMSD criteria are listed (D). (TIF) [file pone.0050186.s007.tif]

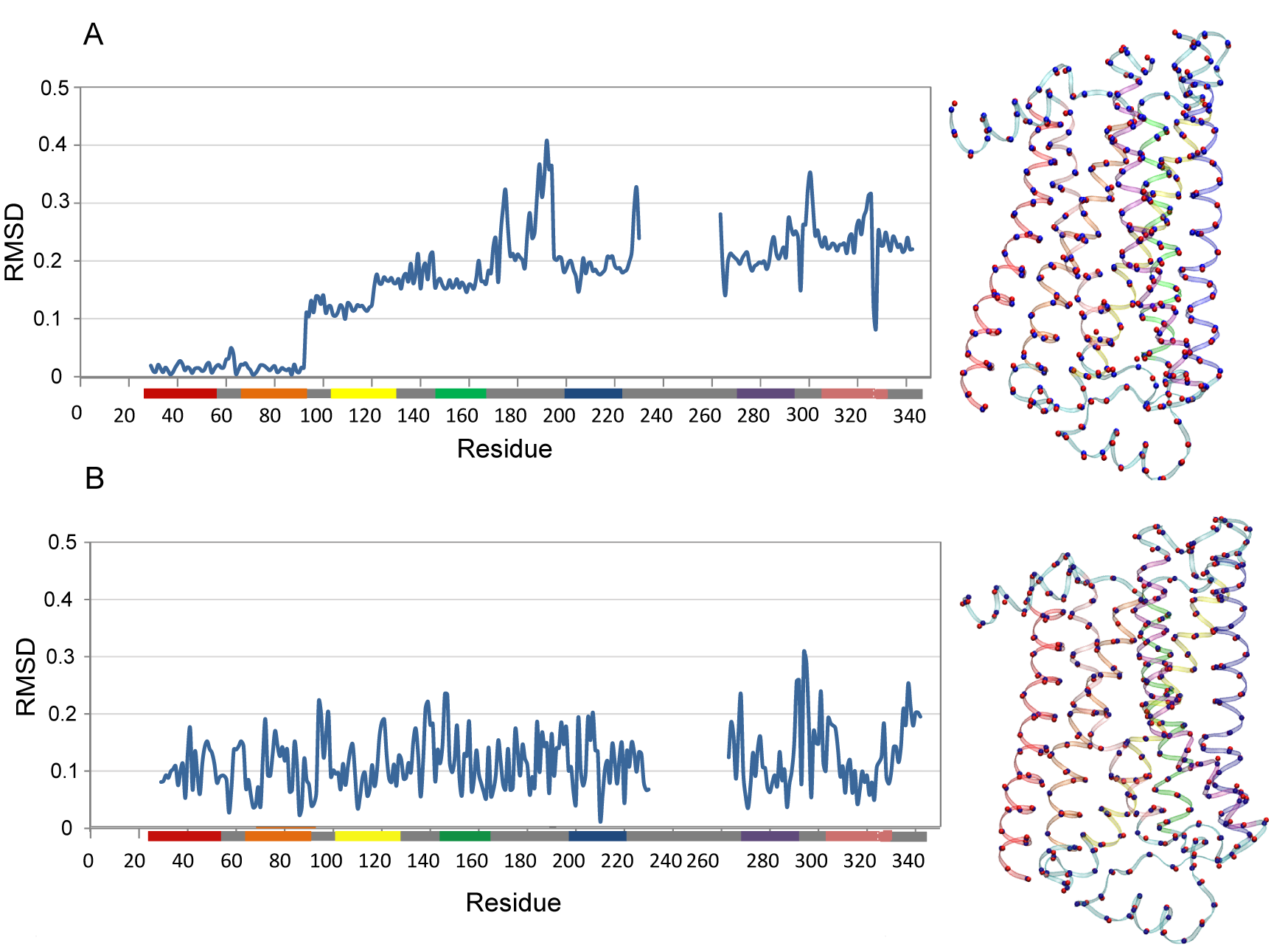

Supplement: Figure S7 — The comparison of the target ANM modes and their corresponding ANM-restrained-MD conformations. The left panels show the RMSD per residue profiles between the ANM modes and their corresponding ANM-restrained MD conformations for mode 8 in minus direction (8M) that forms the complex with salmeterol (A) and for mode 2 in plus direction (2P) that forms the complex with epinephrine. The right panels show ribbon diagrams of 8M (A) and 2P (B) β2AR conformations. The CA carbons of ANM modes and their corresponding ANM-restrained MD conformations are diplayed as red and blue spheres respectively. The helices are colored in accord with the rest of the manuscript. The color code of the helices that are used in the ribbon diagrams of β2AR are also displayed as colored bands at the bottom of the graphs. (TIF) [file pone.0050186.s008.tif]

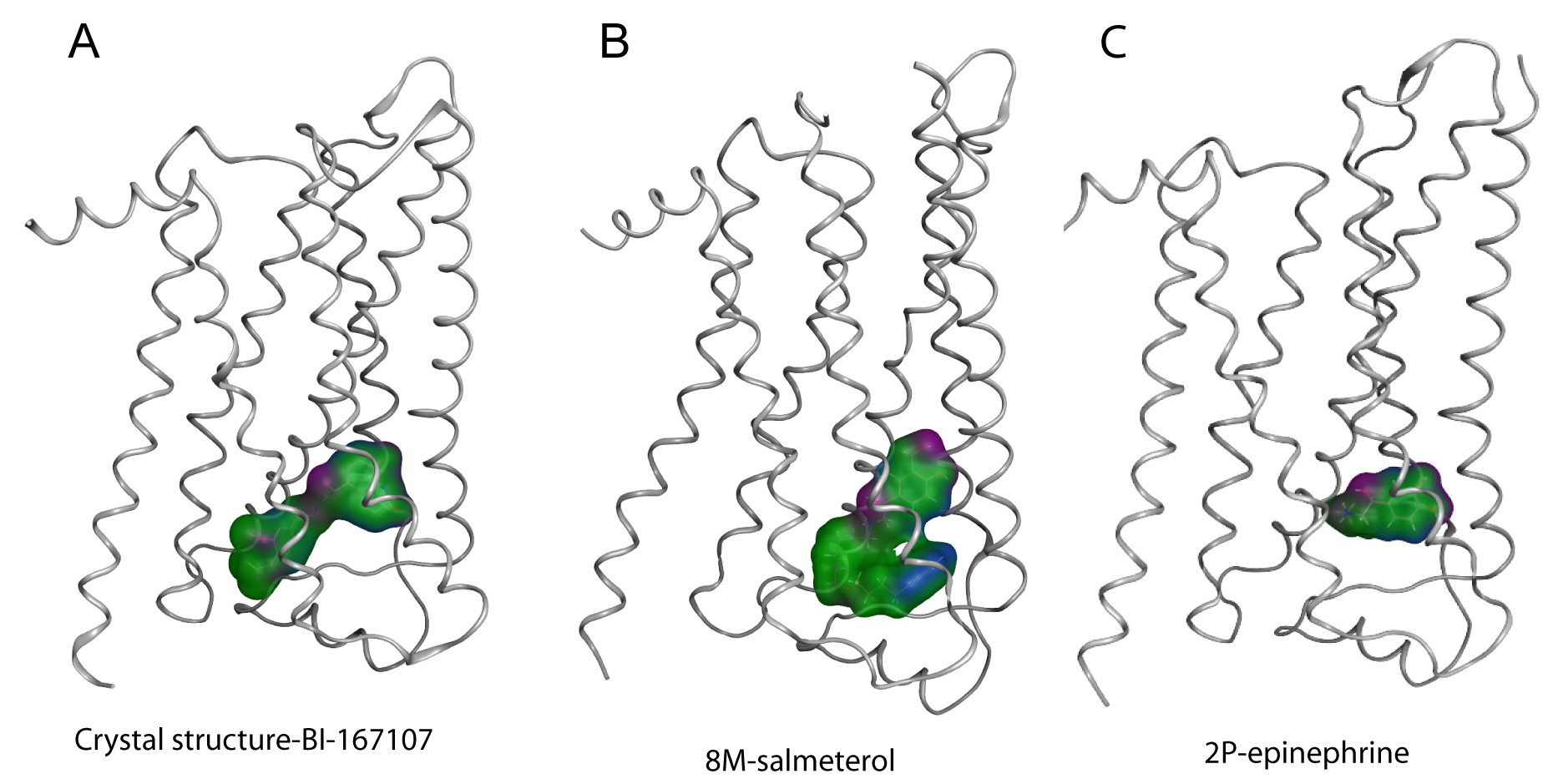

Supplement: Figure S8 — The size and the location of β2AR agonists in the protein-ligand complexes. The head groups of all agonist are located at the orthosteric binding pocket and they interact with the Serine residues at H5 rotated towards the binding pocket. While epinephrine populates only the orthosteric binding site both BI-167107 and salmeterol protrude to the extracellular region. The head groups of all agonist are located at the orthosteric binding pocket interacting with the Serine residues at H5 rotated towards the binding pocket. Ribbon diagrams of β2AR conformations are shown in gray. Molecular surfaces of the ligands are created by using MOE. Green, purple and blue regions of the ligands are hydrophobic, hydrophilic and mildly polar, respectively. (TIF) [file pone.0050186.s009.tif]

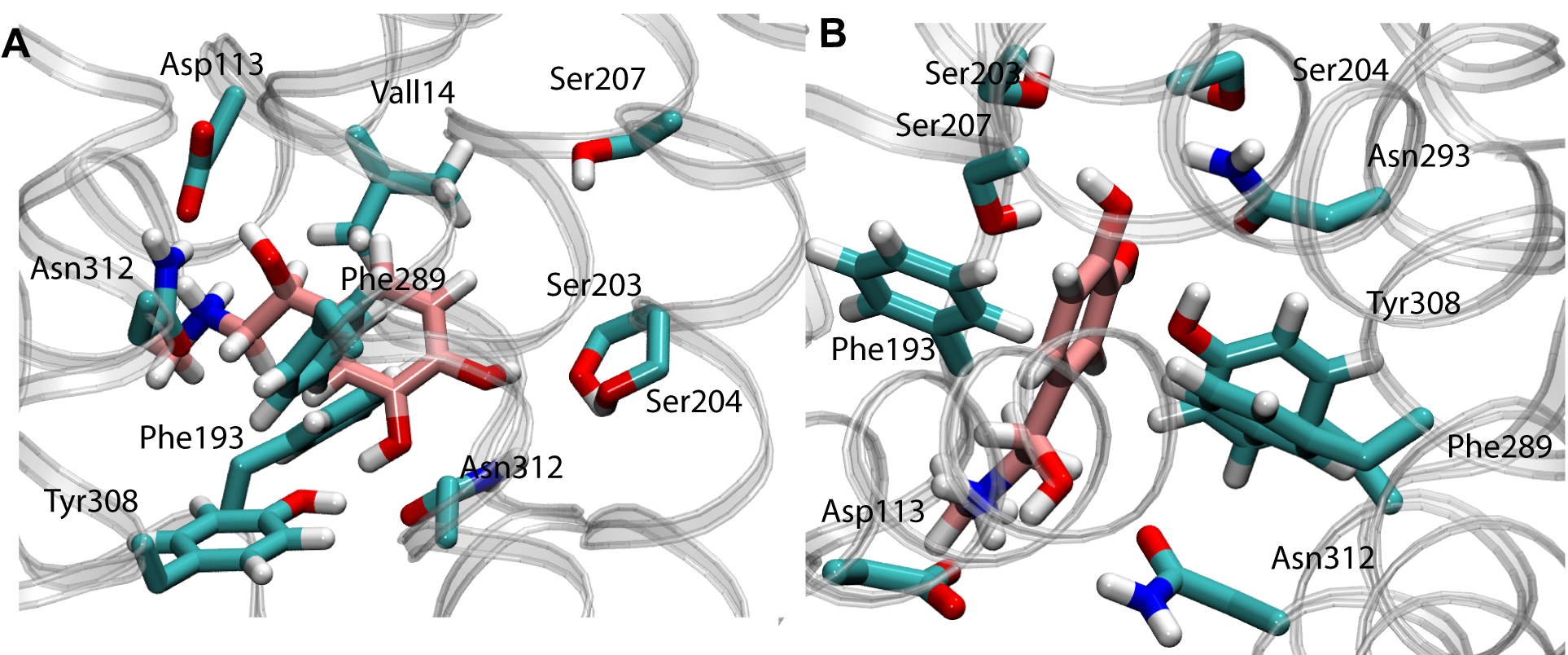

Supplement: Figure S9 — Binding of epinephrine to the active BI-167107 bound form of β2AR. A. The first pose of epinephrine in the BI-167107 bound active structure is located at the orthosteric binding pocket near the experimentally verified ligand binding residues active crystal structure. However, it is not forming any hydrogen bonds with any of these residues such as serines at H5 and Asn113 at H3. B. In the second highest rank pose of epinephrine in the active BI-167107 bound structure it is located at the lower binding site closer to the EC site. At this pose, one of the hydroxyl groups of the catechol is forming a hydrogen bond interaction with Ser203 while the other hydroxyl group is closer to Tyr308 at H7 but not forming any hydrogen bond interaction. Figure S9B shows this pose from a top view from the cytoplasmic site of β2AR. The catechol ring of epinephrine is located between Phe193 at the EC loop 2 and Phe289 at H6 forming π-stacking interactions with both of these residues. Asp113 at H3 and Asn312 at H6 are at the closer vicinity of the β-hydroxyl and amine groups of epinephrine. The epinephrine molecules in complex with the active BI-167107 bound structure are shown in pink color. (TIF) [file pone.0050186.s010.tif]

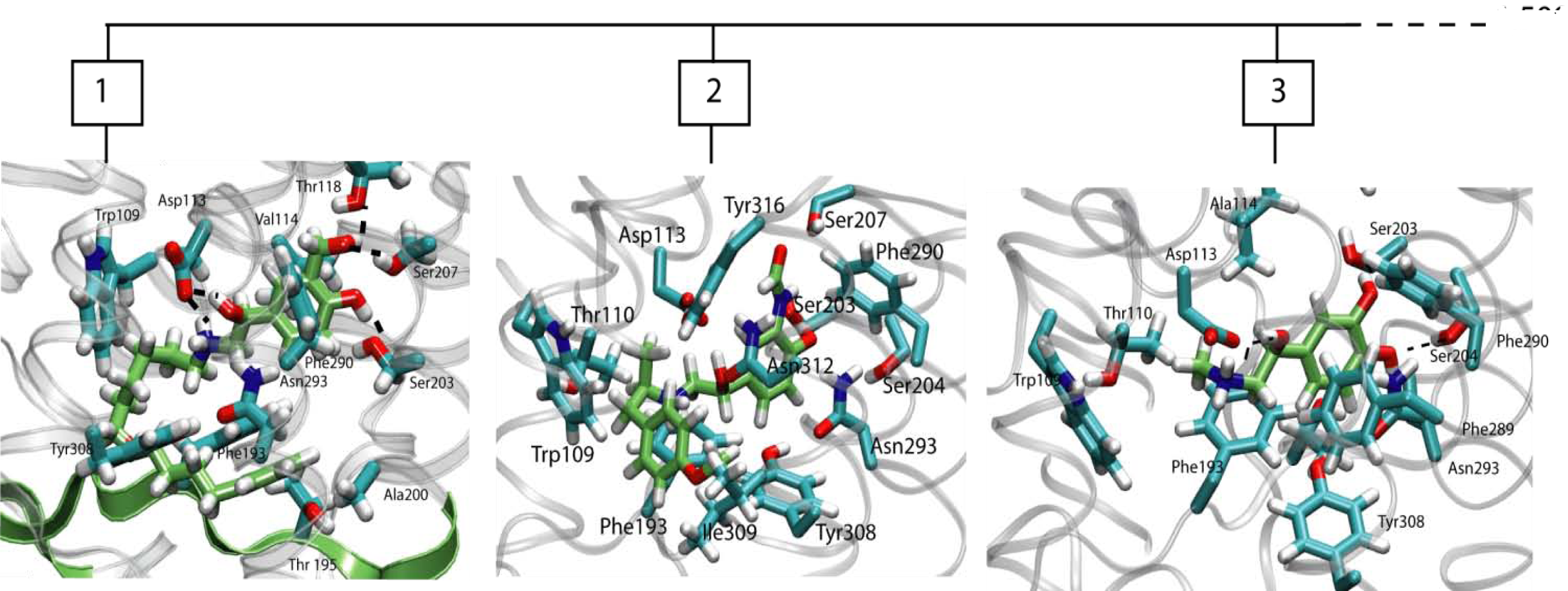

Supplement: Figure S10 — Binding of modes salmeterol, R, R formoterol and epinephrine to β2AR. ANM-restrained MD conformations that accommodate β2AR agonists with experimentally verified interactions are shown. The ANM-restrained MD conformations-ligands complexes are with salmeterol, R, R formoterol and epinephrine are shown in the left-, center- and right panels, respectively. The side chains of the residues that are 3.5 Å of the ligands are displayed. Coloring of the atoms are the same as in Figure 4. The EC2 that interacts with salmeterol is shown in green in the first panel. All β-hydroxyl and protonated amine groups of the ligands are forming hydrogen bonds with Asp113 at H3. The hydroxyl groups of the aromatic rings of all ligands are forming hydrogen bonds with Serines at H5. Epinephrine binds to the orthosteric binding site slightly closer to the EC site compared to the larger ligands, salmeterol and R, R formoterol. (TIF) [file pone.0050186.s011.tif]

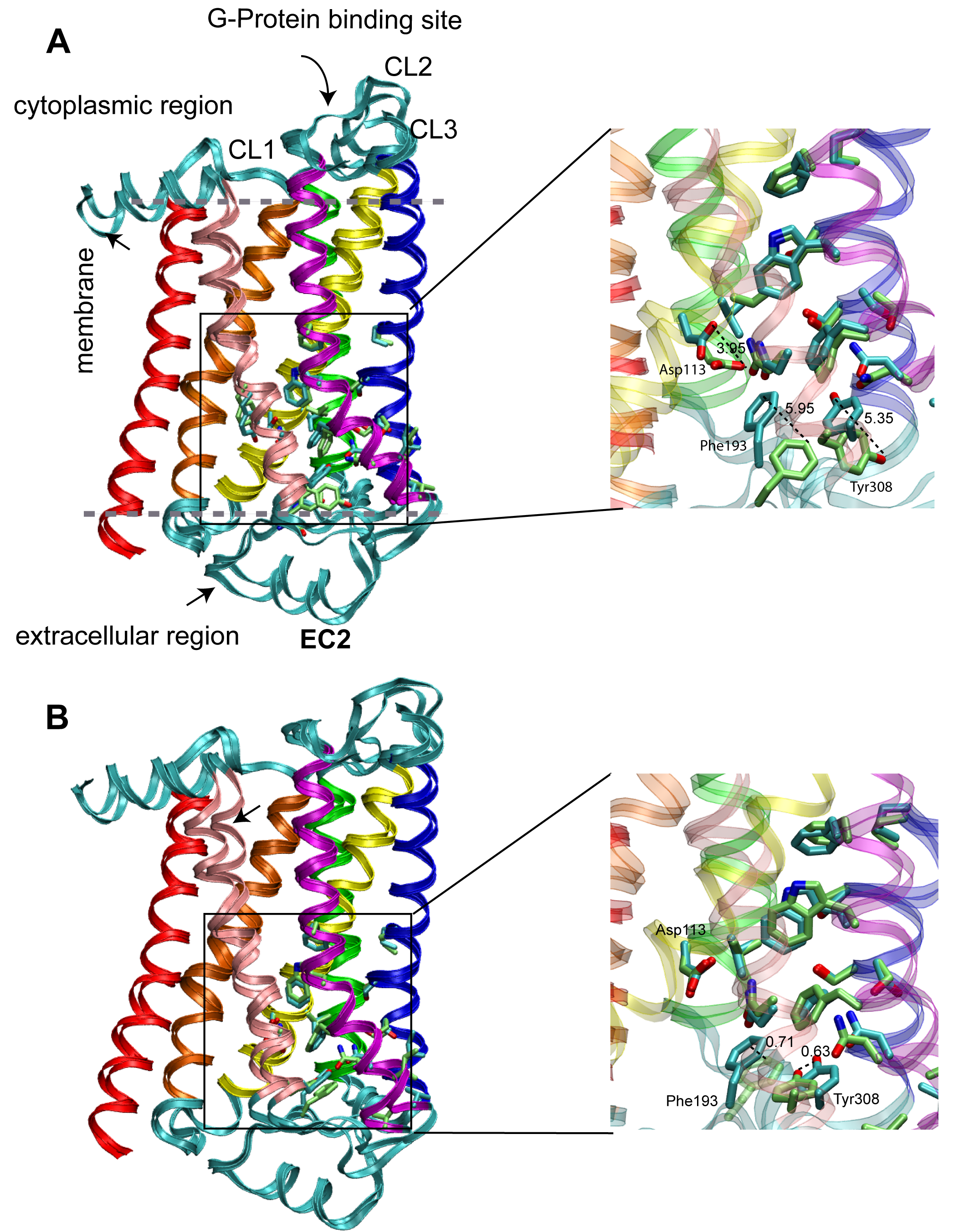

Supplement: Figure S11 — The Comparison of the millisecond scale MD simulation snapshots with the inactive crystal structure of β2AR. The left panels show the ribbon diagrams of the apo (A) and carazolol (B) bound snapshots that are superimposed to the inactive carazolol bound crystal structure. The most pronounced conformational changes are denoted by the arrows. The right panels show the corresponding binding sites of these snapshots compared to the crystal structure. The carbon atoms of the MD snapshots and the crystal structure side chains are colored cyan and green, respectively. The rest of the atoms and the helices are colored in accord with the rest of the document. (TIF) [file pone.0050186.s012.tif]

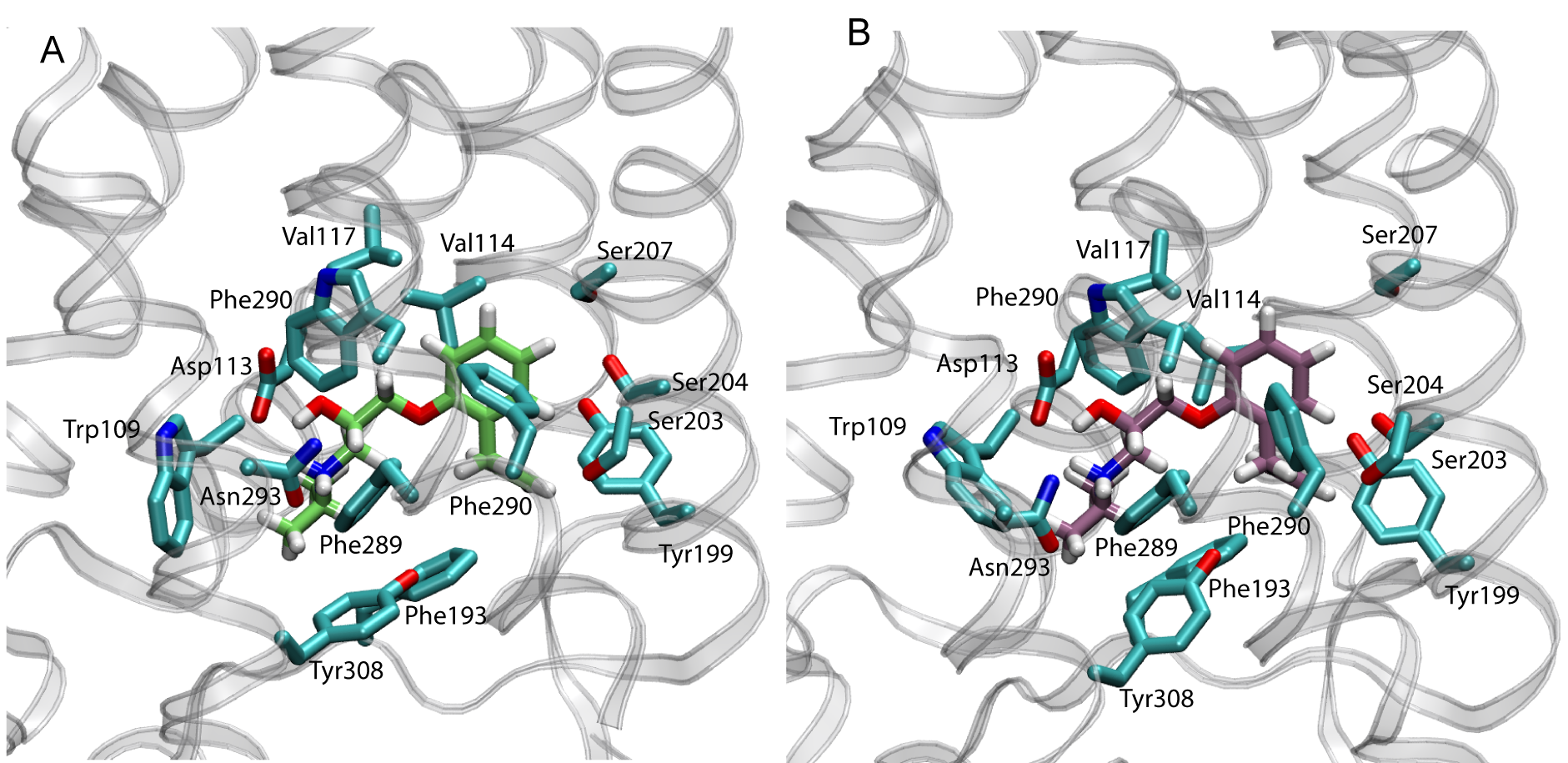

Supplement: Figure S12 — Alprenolol in ANM restrained MD conformation and in the crystal structure complex. Binding residues of alprenolol in ANM-restrained-MD conformation (A) and in the crystal structure (B). Carbon atoms of alprenolol in ANM-restrained-MD conformation (6P) and the crystal structures colored green and pink, respectively. The heavy atoms of the residues that are in the 4.5 A of alprenolol are displayed and labeled. Both structures have the same residues in the vicinity of alprenolol and display similar orientations. Unlike agonists of β2AR alprenolol does not contain hydroxyl groups that interact with the Serine residues at H5. In both structures Ser203 and Ser207 are not rotated towards the binding cavity of β2AR. Instead, the head group of alprenolol is stabilized at the binding site with the π-stacking interaction with the so-called rotamer toggle switch, Phe290 at H6. The rest of the atoms are colored in the same pattern as in the rest of the manuscript. (TIF) [file pone.0050186.s013.tif]

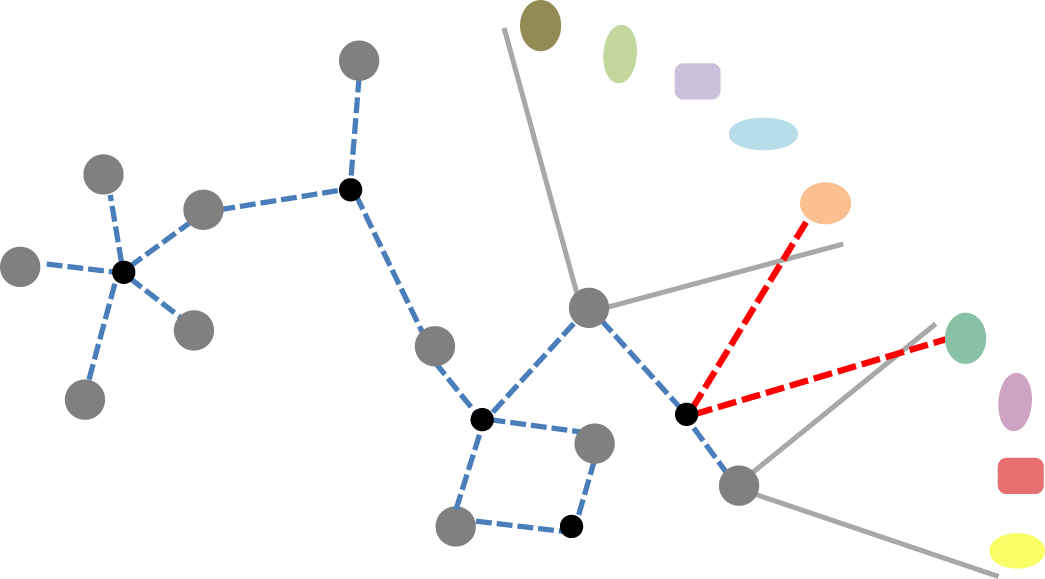

Supplement: Figure S13 — Refining drug target network by assessing protein motion. In current drug target network representations drug targets (gray circles) are interpreted as single entities connected through drugs (black circles), and the particular biologically relevant protein conformations that a drug preferentially binds are ignored. However, to fully understand the interactions of drugs to their targets a target should be represented by its different functionally relevant conformations (different colored shapes within grey line enclosed areas). Drug targets that are represented by single structures are connected to drugs by blue dashed lines. The target conformations that preferentially bind a drug are connected by red dashed lines. (TIF) [file pone.0050186.s014.tif]
